# Supplementary material for: Role of root exudates on assimilation of phosphorus in young and old Arabidopsis thaliana plants
Source: PLoS One. 2020 Jun 3;15(6):e0234216. doi: 10.1371/journal.pone.0234216 (PMC7269232; doi:10.1371/journal.pone.0234216)
Supplement: S1 Fig — (A) 456 compounds detected using GC-MS are plotted on the graph. PCA show dissimilarity among group of metabolites in the seedling stage at different fertilization levels: 25% (light green), 50% (light blue), 100% (green); vegetative stage: 25% (purple), 50% (pink), 100% (blue); and bolting stage: 25% (brown), 50% (olive), 100% (orange). (B) Data reduced to 201 annotated compounds with proper identification. PCA of compounds grouped by phosphate treatments in the seedling stage: 25% (light green), 50% (light blue), 100% (green); vegetative stage: 25% (purple), 50% (pink), 100% (blue); bolting stage: 25% (brown), 50% (olive), 100% (orange). The dotted circle indicates a cohesive group at a given fertilization level. (DOCX) [file pone.0234216.s001.docx]

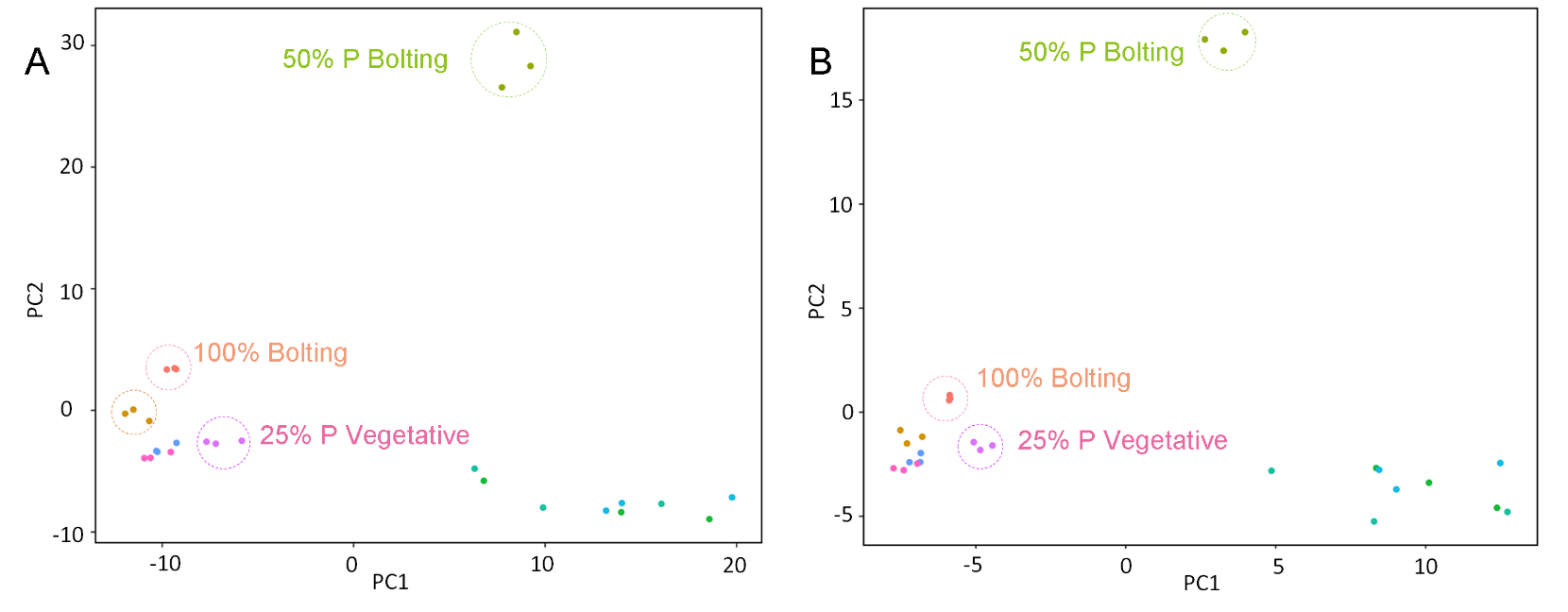


**Supplementary figure 1.** Root exudate compounds diverge in response to plant developmental stage and phosphate fertilization rate. **(A)** 456 compounds detected using GC-MS are plotted on the graph. PCA show dissimilarity among group of metabolites in the seedling stage at different fertilization levels: 25% (light green), 50% (light blue), 100% (green); vegetative stage: 25% (purple), 50% (pink), 100% (blue); and bolting stage: 25% (brown), 50% (olive), 100% (orange). **(B)** Data reduced to 201 annotated compounds with proper identification. PCA of compounds grouped by phosphate treatments in the seedling stage: 25% (light green), 50% (light blue), 100% (green); vegetative stage: 25% (purple), 50% (pink), 100% (blue); bolting stage: 25% (brown), 50% (olive), 100% (orange). The dotted circle indicates a cohesive group at a given fertilization level.
