# Supplementary material for: Role of root exudates on assimilation of phosphorus in young and old Arabidopsis thaliana plants
Source: PLoS One. 2020 Jun 3;15(6):e0234216. doi: 10.1371/journal.pone.0234216 (PMC7269232; doi:10.1371/journal.pone.0234216)
Supplement: S2 Fig — Treatments within plant developmental stages differ from one another, particularly the vegetative and bolting growth stages. Ellipses circle three repetitions of same fertilizer level. Color code correspond to seedling: 25% (light green), 50% (light blue), 100% (green); vegetative: 25% (purple), 50% (pink), 100% (blue); bolting: 25% (brown), 50% (olive), 100% (orange). (DOCX) [file pone.0234216.s002.docx]

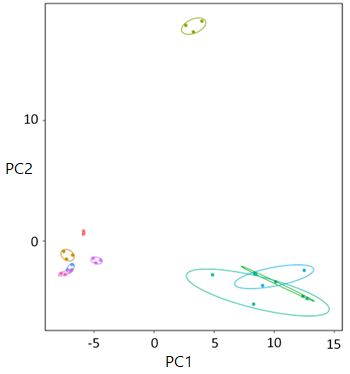


**Supplementary figure 2.** Root exudate compounds grouped by repetitions of fertilizer level. Treatments within plant developmental stages differ from one another, particularly the vegetative and bolting growth stages. Ellipses circle three repetitions of same fertilizer level. Color code correspond to seedling: 25% (light green), 50% (light blue), 100% (green); vegetative: 25% (purple), 50% (pink), 100% (blue); bolting: 25% (brown), 50% (olive), 100% (orange).
