# Supplementary material for: Role of root exudates on assimilation of phosphorus in young and old Arabidopsis thaliana plants
Source: PLoS One. 2020 Jun 3;15(6):e0234216. doi: 10.1371/journal.pone.0234216 (PMC7269232; doi:10.1371/journal.pone.0234216)
Supplement: S3 Fig — Selected compounds based on PCA from vegetative 25% phosphate (A), and vegetative 50% and 100% phosphate (B). (DOCX) [file pone.0234216.s003.docx]

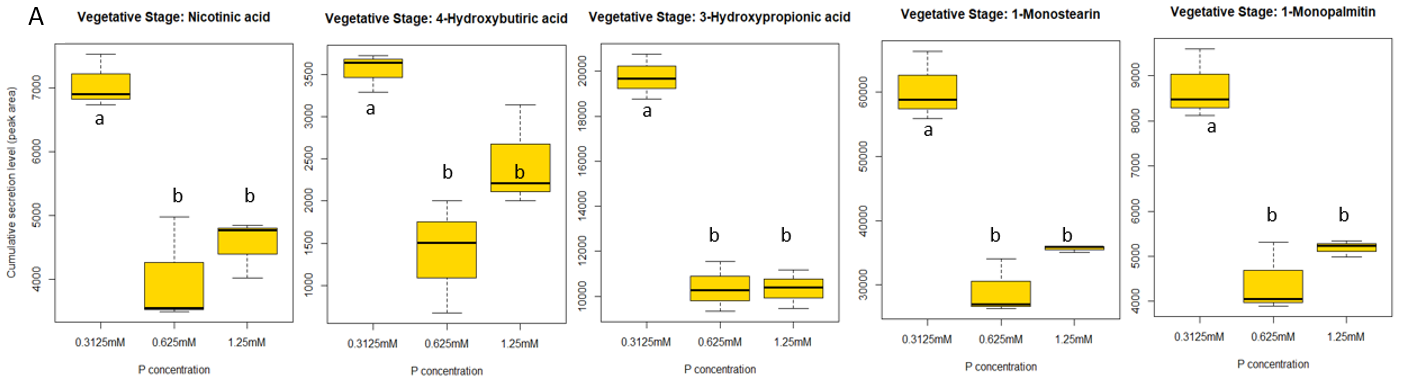


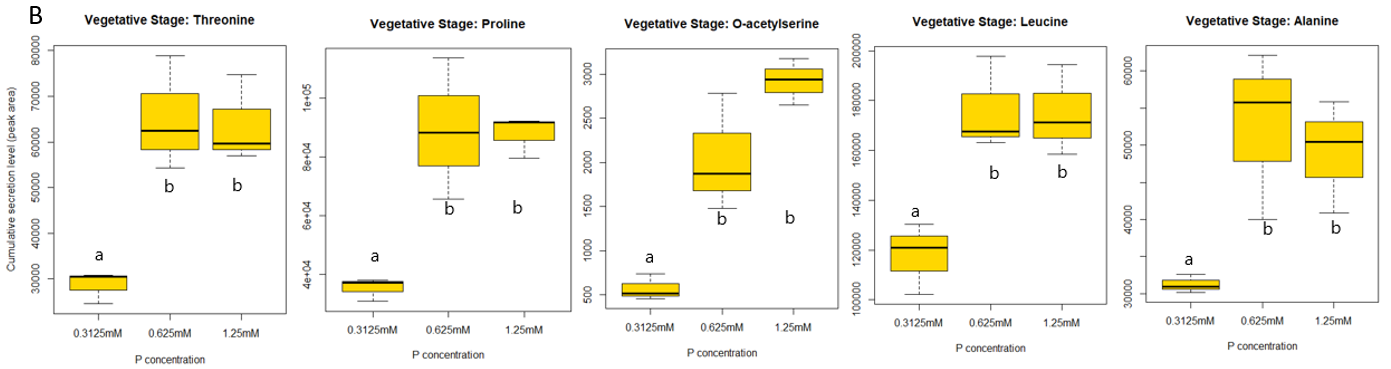


**Supplementary figure 3.** Top 10 compounds showing changes in cumulative secretion levels in the vegetative developmental stage (p<0.05) in response to increasing phosphate addition (0.312, 0.625 and 1.25 mM). Selected compounds based on PCA from vegetative 25% phosphate **(A)**, and vegetative 50% and 100% phosphate **(B)**.
