# Supplementary material for: Role of root exudates on assimilation of phosphorus in young and old Arabidopsis thaliana plants
Source: PLoS One. 2020 Jun 3;15(6):e0234216. doi: 10.1371/journal.pone.0234216 (PMC7269232; doi:10.1371/journal.pone.0234216)
Supplement: S4 Fig — Selected compounds from bolting 25% phosphate (A), bolting 50% P (B) and bolting 100% (C). (DOCX) [file pone.0234216.s004.docx]

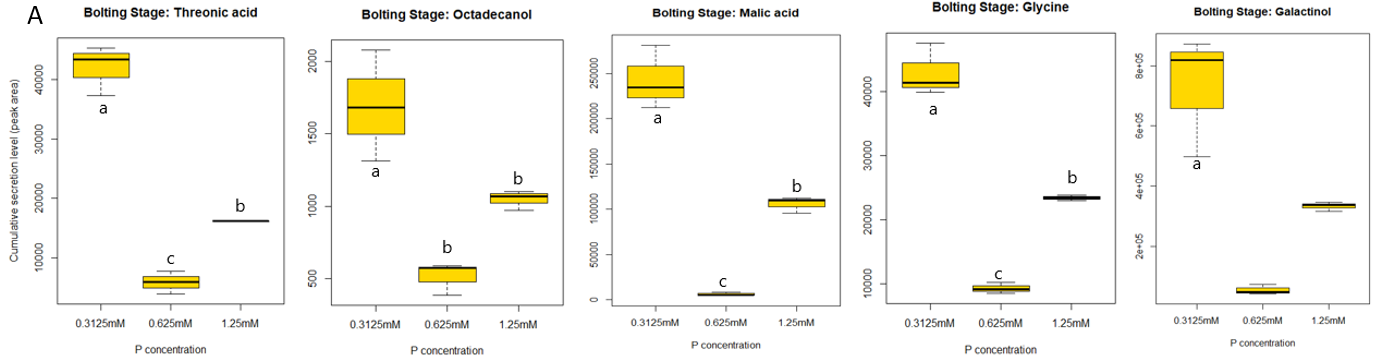


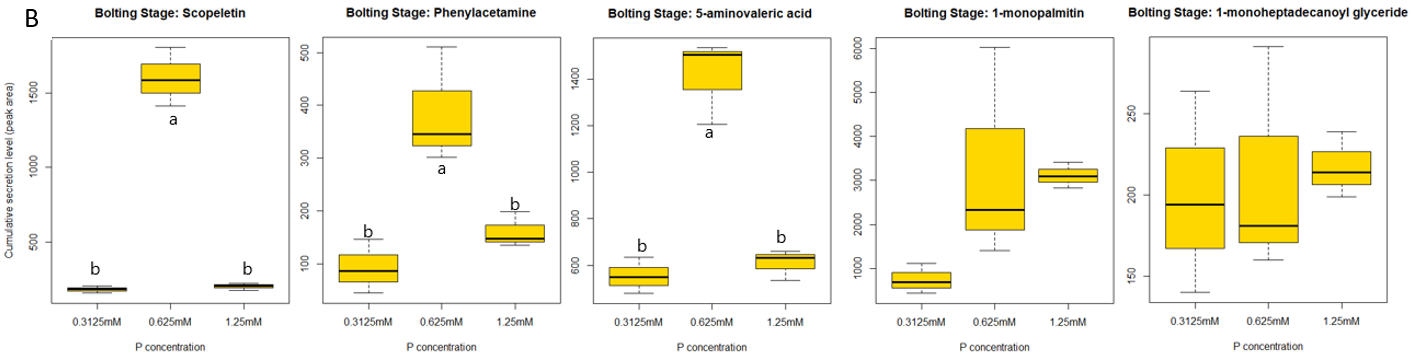


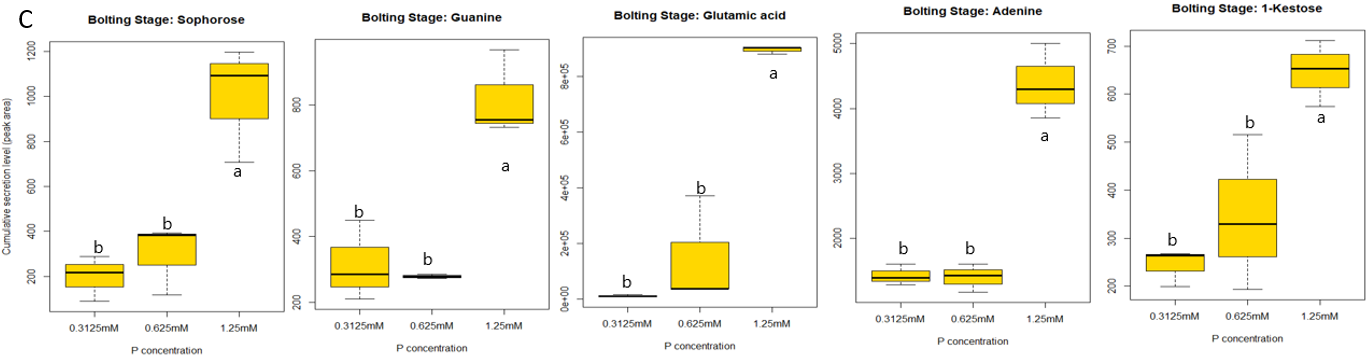


**Supplementary figure 4.** Top 15 compounds showing changes in cumulative secretion levels in the bolting developmental stage (p<0.05) in response to increasing phosphate addition (0.312, 0.625 and 1.25 mM). Selected compounds from bolting 25% phosphate **(A)**, bolting 50% P **(B)** and bolting 100% **(C)**.
