# Supplementary material for: Role of root exudates on assimilation of phosphorus in young and old Arabidopsis thaliana plants
Source: PLoS One. 2020 Jun 3;15(6):e0234216. doi: 10.1371/journal.pone.0234216 (PMC7269232; doi:10.1371/journal.pone.0234216)
Supplement: Attachment — Submitted filename: PONE-D-20-04662_R1TBedts.pdf [file pone.0234216.s006.pdf]

# PLOS ONE

## Role of root exudates on assimilation of phosphorus in young and old Arabidopsis thaliana plants

--Manuscript Draft--

| | |
|---|---|
| **Manuscript Number:** | PONE-D-20-04662R1 |
| **Article Type:** | Research Article |
| **Full Title:** | Role of root exudates on assimilation of phosphorus in young and old Arabidopsis thaliana plants |
| **Short Title:** | Phosphorus assimilation by plant root exudates |
| **Corresponding Author:** | Jorge M. Vivanco<br>Colorado State University<br>Fort Collins, CO UNITED STATES |
| **Keywords:** | Keywords: Arabidopsis thaliana; developmental stage; phosphorous solubilization; root exudate |
| **Abstract:** | The role of root exudates has long been recognized for its potential to improve nutrient use efficiency in cropping systems. However, studies addressing the variability of roots exudates involved in phosphorus solubilization across plant developmental stages remain scarce. In this study, we tested the effect of three increasing phosphorus fertilization levels on roots' exudate composition of Arabidopsis thaliana at three developmental stages (seedling, vegetative and bolting) under sterile nutrient solution-based conditions. The data showed that the total exudates changed in response to the incremental addition of phosphorus starting at the vegetative level. It was found that specific metabolites decreased in quantity to phosphorus supplementation at specific stages of development. Some of those metabolites were tested for their phosphorus solubilization activity, and it was found that 3-hydroxypropionic acid, malic acid, and nicotinic acid were able to solubilize phosphorus under solid and liquid NBRIP media conditions. In summary, our data suggest that plants can release distinct compounds to deal with phosphorus deficiency needs influenced by the phosphorus nutritional status at varying developmental stages. |
| **Order of Authors:** | Hugo A. Pantigoso |
| | Jun Yuan |
| | Yanhui He |
| | Qinggang Guo |
| | Charlie Vollmer |
| | Jorge M. Vivanco |
| **Opposed Reviewers:** | |
| **Response to Reviewers:** | Reviewers responses:<br>Line 1. Title. Your title is difficult to parse. Please rewrite. For example: "On the role of root exudates on the assimilation of phosphorus in young and old roots".<br><br>RESPONSE: Thank you. As suggested, the title was rewritten and now it reads as follow: 'Role of root exudates on assimilation of phosphorus in young and old Arabidopsis thaliana plants<br>Line 26, and elsewhere. Please spell out phosphorus everywhere in the text. Do not use "P". I am aware that many authors use this abbreviation. However repeated use is a poor argument for validity. In fact, abbreviations and acronyms make a text difficult to read. Abbreviations rarely occur in newspapers or fiction. Any time a reader encounters a symbol (P is a symbol for phosphorus), they must translate that symbol into words. Translation takes mental energy away from comprehension. Translation slows down the reader and gives nothing in return. It is one thing to have to write about a chemical with a name that is 38 syllables long. In that case, the name is just as difficult to read as the acronym. However phosphorus is a good word of the English language. In fact, phosphorus literally means 'carrier of light'. Rather beautiful. Spell it |

| | |
|---|---|
| | out.<br><br>RESPONSE: Thanks for the suggestion and wonderful comments particularly related to “carrier of light”. The abbreviation for phosphorus was changed for the full word throughout the manuscript.<br>Line 26. “… have not been conducted…” This wording implies that you have read every paper ever published. Use less extreme wording, such as “…rarely if ever…”<br><br>RESPONSE: Thanks for the suggestion. We have changed ‘have not been conducted’ for ‘remain scarce’.<br>Line 29. You are using the word ‘metabolome’, to refer to root exudates. This is misleading and confusing. The word metabolome refers to all of the metabolites in the cell or organism. However here, you are not measuring the metabolome. Instead you are measuring exudates. These compounds number about a dozen, far less than the threshold for ‘omics’. Please remove the word ‘metabolome’ throughout the paper and instead talk about ‘total exudates’ or the equivalent. For example, the sentence staring at line 29 can read: “The composition of root exudates changed in response…”<br><br>RESPONSE: Thanks. We agreed with the editor. We replaced the word metabolome for total root exudates and/or total exudate profile throughout the manuscript.<br>Lines 29 and 34. You write ‘in vitro’ conditions. What does this mean? Instead say what the conditions are. By the way, typically in vitro implies isolated components, cells or cell fragments. I have never seen in vitro used for whole organisms. Also you write ‘solid’ and ‘liquid media conditions’. Again, be specific, say what the solid and liquid media are.<br><br>RESPONSE:<br>Thank you for the recommendation. To make the distinction, we have replaced the word in vitro for ‘sterile nutrient solution’.<br>The type of media described as solid and liquid is a microbiological growth medium for screening phosphate solubilizing microorganisms known as NBRIP (National Botanical Research Institute’s phosphate) growth media. This information appears in the material and methods. In line 29-34 (now 36) we have specified the type of liquid and solid media and it reads now as: “solid and liquid NBRIP media conditions”.<br>Line 31. What does ‘respond negatively’ mean? If you mean decreased in quantity, just say so.<br><br>RESPONSE: Thank you. Changes were made accordingly and now the sentence reads as follows: ‘It was found that specific metabolites decreased in quantity related to phosphorus supplementation at specific stages of development.’<br>Line 66. Based on long-established rules for scientific nomenclature, the word “Arabidopsis” (Capital A, italic font) means the genus. No matter how many illiterate molecular biologists make this mistake, rules of taxonomic nomenclature remain in force until the international committee decrees otherwise. Longstanding practice allows “arabidopsis” (lower case a, Roman font) as the common name of our friendly lab weed; however, many journals will auto-correct this by adding a capital and italics. I am not sure about PLoS One. Thus, either use ‘arabidopsis’ and hope the journal lets it stand, or use A. thaliana(italics) (but Arabidopsis thalianaat first mention).<br><br>RESPONSE: Thanks for the observation. Arabidopsis thaliana was first mentioned in the abstract and ‘Arabidopsis’ word was replaced by A. thaliana throughout the manuscript.<br>Line 83. This should read “Arabidopsis thalianaL. (Heynh) wild-type Columbia seed…” It is customary in the Material and Methods to give the full Latin binomial along with the taxonomic authority. And unless you sequenced your Columbia line, you should drop the “0”.<br><br>RESPONSE: Thanks for the observation. The sentence was modified and now it reads as follows: ‘A. thaliana (L.) Heynh wild-type Columbia seeds’.<br>Line 84, and elsewhere. Do not use circle-R, or TM, or other commercial symbols. Those marks exist to protect consumers (not manufacturers) from fraud. Thus if you sell a product that you say contains X circle-R, you are defrauding the consumer if you substitute something cheaper for X. There is no such issue here. In fact you are not selling anyone anything (you are paying). |

| | |
|---|---|
| | RESPONSE: Thank you. Changes were made accordingly.<br>Line 98. I don't understand the meaning of the word 'power' here.<br><br>RESPONSE: Thank you. The word power is not necessary. It was deleted.<br>You embedded the figure legends into the results text but not the figures. I think this is awkward. Please put the figure legends with the figures, preferably at the end of the text. Also for the principal component analyses, please simply call your x and y axes "PC1" and "PC2" and put the percentages of explained variance in the figure legend.<br><br>RESPONSE: As suggested, figures and legends are together in a separate file uploaded right after the text.<br>All principal component analyses have been modified and the explained variance is now shown in the legend.<br>In figure 3A, the names of some compounds are capitalized but others are not. And "control" is in all caps. This is bizarre. I suggest that none of them should be capitalized, but whatever you do please do the same for all. And note that if you choose to capitalize the first letter, this should be done even for those compounds that start with a number (e.g., "5-Aminovaleric acid"). Also, please put the name of the horizontal axis below, next to the numbers (or put the numbers above, next to the name; the point is, the numbers and the name belong together.<br>Also please remember that the abbreviation for liter is L (not l). And to always put a space between the number and the unit (except when the unit is the degree sign or the percent sign).<br><br>RESPONSE: Thanks for the observation. The first letters of all compounds have been capitalized, and the horizontal axis is now next to the numbers. The abbreviation L and the space between the number and the unit was also modified as suggested.<br>In Figure 4, there are problems with the y-axes. Many of them have huge numbers. But no units. I think peak area is arbitrary so you could use 1, 2, 3. If it is needed to compare the quantity between states, then you could have the smallest numbers be 1, 2, 3 and the other states relative to that. Also many of the graphs do not start at zero. But some of them do. This is misleading. They should all start at zero. The names of the axes could be in a larger font.<br><br>RESPONSE: Thank you for the observation. The y-axis values show the magnitude of the cumulative secretion level of the compound per each stage. Those values are different based on the compounds. Thus, standardizing the values for all compounds will make some compounds hard to asses due to low levels. This set of plots aimed to provide to the reader with a visual aid to see significant differences by compound between the phosphorous treatments.<br>We think keeping the peak area unit as it is will help the reader to determine the degree of change (of the compound) in response to P amendment as well as to locate the compound that was the most affected by phosphorus amendment.<br>With respect to graphs not starting at zero: the compounds with statistical significance are properly labeled which will help the reader to see which of those secretion levels are highly responsive to phosphorus addition. We do not think this is misleading and we would rather keep it as it is.<br>Reviewer #1: Two minor and three major critics<br>minor:<br>1. Root exudates depending on plant develpment were reported by Keerthisinghe et al. 1998, Plant Cell Environ., 21, 467- 478 and by Neumann et al., 1999, Planta, 208, 373-382.<br><br>RESPONSE: We appreciate your valuable comments. Both papers are very relevant for our work, and we have included references about that work in our manuscript. We modified the abstract to state that: 'However, studies addressing the variability of roots exudates involved in phosphorus solubilization across plant developmental stages remain scarce'. Instead of saying that these studies 'have not been conducted'.<br>Having said this, in contrast with Keerthisinghe et al. (1998) and Neumann et al., (1999), our study showed effects of P supply on non-proteoid plant-roots such as Arabidopsis.<br>Neumann et al., (1999) support our findings showing that phosphorus mobilizing compounds such as citric acid was predominantly restricted to mature root clusters in |

Powered by Editorial Manager® and ProduXion Manager® from Aries Systems Corporation

the later stages of P deficiency. However, they conclude that this mechanism is 'predominantly confined to proteoid-roots' which is not the case for Arabidopsis as shown in our study. We have incorporated this to the discussion section.

2. L 278 ff. Misleading since P and carboxylate concetrations must be clearly separated.

RESPONSE: Thank you for the observation. We have rewritten this sentence to better describe how cumulative secretion of different carboxylates vary depending on the treatment (phosphorus level) and to make the distinction between phosphorus and carboxylates. Now the sentence reads as follows: 'Cumulative secretion of malic acid was reduced significantly from 0.3125 to 0.625 mM phosphorus treatments, and then incremented its cumulative secretion for the highest phosphorus treatment (1.25 mM). However, secretion levels of malic acid for the two highest phosphorus treatments (0.625 and 1.25 mM) were below the value for the lowest phosphorus rate (0.3125 mM) (Fig 4A).'
major:
1. Misleading: P starvation response 50 µM P. Cultivation in non buffered systems, agar or solution means that a solution threshald is only valid for a special volume. In soil P is buffered and P starvation values are below 2 µM P , see Föhse te al., 1988, Plant Soil, 110, 101- 109.

RESPONSE: Thank you for the clarification. As per your suggestion, we clarified that plant starvation response to phosphorus is activated at values below 2 µM P. We have added Föhse et al., (1988) reference to the manuscript and the sentence now reads as follows: 'The three phosphorus levels used in this study did not induce the plant starvation response, which is generally activated at values below 2 µM of phosphorus in the soil solution [23].

2. Decisive for the solubilizing ability of root exudates is the relation between soil solid P and the quatity of exudates (see in detail Gerke, 2015, cited in the ms). Solution concentrations are not an appropriate measure of exudate efficiency. Mesurement of pH changes as a central factor of P mobilization from Ca-P forms?

RESPONSE: We appreciate your valuable comment. We are aware that a combination of factors including carboxylate efflux, the accumulation of carboxylate in the rhizosphere and the chemistry of phosphorus mobilization in the soil phase affect the solubilization ability of root exudates (Gerke, 2015). We acknowledge that we did not take into account these variables, because our study was entirely done in liquid culture to test solubilization ability of the selected compounds, not necessarily efficiency which would require the measurement of abovementioned factors. However, we have added a paragraph in the discussion section that address pros and cons of root exudate studies in liquid conditions (our study).
The paragraph added reads as follows: 'Lastly, root exudates from liquid culture systems allow the determination of exudation rates unaltered by the soil matrix or microbial decomposition if performed under sterile conditions as we did in this study (Oburger and Jones, 2015). However, the quality and quantity of the root exudate profile may be impacted by the nutrient solution culture method (also known as hydroponic methods) (Oburger et al., 2013). Soil-hydroponic hybrids methods for root exudation collection are not exempt of potential physical/physiological perturbances. Thus, sterile nutrient solution culture methods remain especially important to assess temporal dynamics of root exudates.'

3. The relevance of the results with respect to P solubilization is questionable since the P form is rather unrepresentative and the exudate quantity is rather high.

RESPONSE: Thank you for your observation. It would be helpful if the reviewer elaborates on his comment. Why are the P form is unrepresentative? Is it because calcium phosphate is not necessarily predominant in all agricultural soils? Calcareous soils constitute over one-third of the world's land area. Why you consider the root exudate quantity as high, relative to what? Root exudates concentration varies a lot across P levels, developmental stage and on a per compounds basis in our study.
Reviewer #2: General comments:

Powered by Editorial Manager® and ProduXion Manager® from Aries Systems Corporation

The manuscript “Phosphorus assimilation effects by root exuded compounds across plant developmental stages” characterises root exudates across growth stages and P-levels for Arabidopsis. Interesting contrasts were found. The authors select some important exuded compounds and go on to test their ability at solubilising P in soil-analogues individually and together. This was a well written manuscript, with well thought out experiments, executed well leading to interesting results. I really enjoyed reading this manuscript, I learnt a lot that is relevant to my work, and appreciated the work that went into it. I have some general comments that I think would improve the presentation and some of the discussion.

1) I think in the materials and methods the presentation of the PCA is sometimes over complicated, and could be made simpler. My detailed comments below would address this.

RESPONSE: Thanks for the valuable observations. Detail corrections regarding the PCA plots have been addressed in each of your comments below.

2) The presentation of the PCA results could be improved. I think showing all the data with no groupings used on the PC-axis (with different colours and marker styles and legends!) would make it much clearer an. This could come at the expense of some of the plots where treatments are grouped. Also make it clear if a PCA is done per grouping or not. Again, many of my detailed comments try and address this.

RESPONSE: Thanks for the valuable observations. Detail corrections regarding the PCA plots have been made and addressed in each of your comments below.

3) The discussion would be improved by quickly pointing out the agricultural/ecological significance (or lack of) of the results. For example 7mM of acids order of magnitude more than what is found in the rhizosphere. See the Eva Oburger papers I referenced. Hence the solubilisation seen here might not appear in the field. One of our group's papers found that P solubilisation by a single root exuding citrate at a realistic rate actually made no difference to P uptake by the root https://doi.org/10.1007/s11104-019-04376-4 (dont feel you have to reference this). It also might be worth pointing out how similar NBRIP media is to soil, solubilisation in this media might not be the same as soil. Also see my detailed comment regarding your proposed mechanism number 1.

RESPONSE: Thank you for the observations. We have added a paragraph that discusses the relevance and ecological significance of organic acids as phosphorus mobilizers and the relevance of the root exudate analysis in liquid cultures.
The new paragraph reads as follows: ‘It has been estimated that organic acids constitute 5 to 10 % of the total organic carbon in the soil solution. The concentration of organic anions measured in the soil solution usually range from 100 nM to more than 580 uM in the rhizosphere of cluster roots (Jones, 1998). However, millimolar concentrations of organic anions are likely required in the soil solution to effectively increase soluble P concentration especially in calcareous soils (Strom et al. 2005; Ryan and Jones, 2001). Strom et al. (2005) tested three organic acids (citrate, malic and oxalate) and a wide range of concentrations (1 mM to 100 mM) to evaluate its effects on the mobilization of phosphorus in calcareous soil. The results showed that the phosphorus mobilization of the tested compounds had a low efficiency and its effect varied depending on the type of organic acid, compound concentration, and pH. Further, due to the low phosphorus mobilization efficiency of those compounds it is still argued if the benefit of releasing large amounts of organic acids into the soil will exceed the cost of carbon lost by the plant, which can be seen as an unnecessary trade-off (Strom et al. 2005). However, low efficiency organic acids can be particularly important in phosphorus mobilization for calcareous soils with a limited phosphorus availability for plants. Finally, our evidence supports the above-mentioned hypothesis, that plants release a combination of compounds with different phosphorus-solubilizing efficiencies, at specific stages of growth, to deal with particular phosphorous needs.’

Sorry, I have a lot of detailed comments, however many of them are pointing out the same thing and many are complimentary. I hope they aren’t too hard to deal with.

Detailed comments:
L25: roots->root

RESPONSE: Thank you. Please see correction in line 28.
Abstract is clear and well written.
The paragraph starting line 67 doesn't segway smoothly into that starting on 73 because root exudates have been shown to be less important for good P conditions (not to say that the paragraph on L67 isnt useful introductory information). Calling back to the 'legacy P' argument here would make it smoother.

RESPONSE: Thank you for the observation. We considered your suggestion and have improved the transitioning sentence between the two paragraphs. Now it reads as follows: 'Due to the variation of phosphorus demand during the plant's lifetime, it becomes necessary to fully understand fluctuations of root exudates as a means to solubilize the phosphorous present in the P legacy of the soil.'

L90: 'gradient' isn't the best word here. It makes it seem like the growth media each plant is grown in has a gradient of P conditions in it. Is 'The low P conditions used in this study….' Better?

RESPONSE: The authors used gradient to refer to the three concentrations of phosphorous used in the study. For clarity, we changed it to: 'The three phosphorus levels used in this study did not stimulate the plant starvation response, which is generally activated around 2 µM of P [23].'
L129: '…which compounds had the highest correlation with these components through the magnitude of the variance'. 'correlation' is unclear here. Do you mean which two original basis vectors (compounds) explained the most variance? This would be an easier way of explaining it.

RESPONSE: Thank you for the observation. No, in this sentence (now line 140) we refer to the magnitude of the loadings of the compound (variance of the compound secretion level) not to the PCA explained variance. We elaborate on the meaning in more detail in the two following sentences in the manuscript.

L133: 'The largest…' Is it not that these compounds explain the most variance? Its best to write these in terms of what percent of the original variance they explain (before dimension reduction).

RESPONSE: Here we refer to the variance of the individual compounds based on the secretion level (loadings). Compounds with the largest loadings drive the PCA variance and therefore were selected for further testing. Please see line 131 to 146 for more detail.

L134: differential isn't the right word here.
I think the end of this paragraph can be explained in a simpler way by saying: "We determined which compounds explained the most variance for each P level and growth stage. The contrasts in these 'most principle compounds' over growth stage and P level indicated differences in exudation depending on these factors" Or something like this.

RESPONSE: Thank for your suggestion. We have modified the sentence for clarity and now it reads as follows: 'this method allowed us to determine which compounds explained most of the variance across the fertilizer levels and for each of the plant's growth stages.'

L144: This needs more explanation this is quite a large range, how did you 'adjust' the concentrations? The reader needs to know how this is done to interpret the results. Did you take concentrations from these references to calibrate your values? Due to the variability in reported exudate concentration due to sampling technique (https://doi.org/10.1016/j.rhisph.2018.06.004) 'exudate concentration' is relative to the study. Probably needs a mention in the discussion.
Side note: Could be worth mentioning that you didn't adjust the concentrations for the PCA, when I first read it I thought you had.

RESPONSE: Thanks for your observations. As stated in the text, we used a range of concentrations reported in the literature as a reference to select the concentration of

| | |
|---|---|
| | the compounds tested in this study. The word ‘adjust’ has been replaced by the word ‘select’ for clarity.<br>As you well pointed out, the range of concentrations of this compound is wide. We cited 3 papers that support that the concentrations used in our experiments are present in the rhizosphere (Jones, 1998; Veneklaas et al., 2003 and Ström et al., 2005). In addition, we have added a paragraph in the discussion where we elaborate on the concentrations of compounds selected for this study (please see previous answer to your comment) and included the paper that you suggested: Oburger and Jones, 2018.<br><br>L152: What concentrations did you add to test the P solubilising ability of the compounds? Were they comparable to rhizospshere concentrations? Knowing this puts this into an ecological context for the reader. Is the visual inspection for solubility standard? Add a reference if so. Otherwise explain the reasoning.<br><br>RESPONSE: Thank you for the comments. We direct the reviewer to the methodology sub-section (149-160) where the rationale for selecting the concentrations to test P solubilization and the actual concentrations are stated; as well as the paper showing the efficacy of the qualitative technique used in our study. Please see reference number 30 in the reference list (Nautiyal et al., 1999. FEMS microbiology Letters, 170(1), 265-270).<br><br>Table 1: were they diluted to 100mM before PCA? Would this effect PCA? (see my side note above). 100mM is a really high concentration for most root exudates in the rhizosphere no? I know for most organic acids and amino acids I would expect micro molar concentrations in the rhizosphere, see https://doi.org/10.1016/j.envexpbot.2012.11.007<br><br>RESPONSE: Thanks for the observation. Indeed, PCA show total root exudates as a response to phosphorus levels. We agree, 100 mM is in the higher end of the concentrations. This concentration was used only in the qualitative experiment (Petri dish). Several lower concentrations were used as well, and we decided to use 100 mM for this qualitative analysis which aimed to provide an initial visual tool to narrow down a long list of compounds that could solubilize P. Please note that we reduced this concentration to 7 mM and the compounds still showed phosphorus solubilization.<br>L171: again this a high concentration for the rhizosphere, I would expect a discussion on the ecological relevance later.<br><br>RESPONSE: This question was addressed above.<br><br>L181: I think ‘cumulative’ effect is better than ‘additive’. Additive makes it seem like you could add up the effects individually to determine to summed effect.<br><br>RESPONSE: Thank you. We replaced the word additive for the word ‘cumulative’ to better describe the meaning.<br>L200: unclear what ‘distribution’ means here. After looking at FigS1 we see it’s the groupings after plotting on the first two principle components. This needs to be made clearer. I think the author means the separation of growth stage and P treatment by PCA persists after throwing away the non-annotated compounds. Which implies the non-annotated compounds explained little of the variance, which I believe justifies the author’s conclusion. Were the principle components determined twice i.e. before and after throwing away the non-annotated compounds? FigS1 could use a legend saying what the other colours are.<br><br>RESPONSE: Thank you. For clarity, the word distribution has been replaced by the word “grouping”. In addition to indicating that the PCA was conducted with the data after removal of the non-annotated compounds, we have modified this sentence and now it reads as follow: ‘The variability in our data after subtracting the non-annotated compounds was analyzed using a principal component analysis (PCA) where variability of component 1 (PC1) accounted for 29.8%, while component 2 (PC2) accounted for 21.7%.’<br>PCA from figure 1 is identical than supplementary figure 1. Both depict the overall dissimilarity of compound groups across phosphorus levels and growth stages. Both figures show detailed information in the legend now. |

Powered by Editorial Manager® and ProduXion Manager® from Aries Systems Corporation

Fig 1A also needs a legend.

RESPONSE: Thank you. Figure 1A has now a legend with the suggested information.
In Fig 1A did you group by developmental stage, do PCA then plot? If this is the case, the % variance explained by the PCs reported in line 203 isnt correct.

RESPONSE: Thank you for the comment. For figure 1 as well as for supplementary figure 1, no grouping was performed before constructing the PCA. The complete data set from three developmental stages and 3 levels were plotted and analyzed at the same time without previous modification. As suggested, the explained variance of figure 1A was added to the legend. For more detail, please see line 132-144.
Same goes for Fig 1B but by P level? The different percent of variance explained by components suggests this is the case. Need legend for Fig 1B also.

RESPONSE: In figure 1A, we present the three plant stages and three phosphorus levels (all data). Figure 1B presents data for three phosphorus levels within the vegetative stage only, and figure 1C presents the three phosphorus levels within the vegetative stage only. The explained variance for each PCA has been added to the figure legend.
I would use colours to indicate developmental stage and stars/dots/crosses to indicate P-levels (or vice versa). This and a legend would make fig 1 a lot easier to understand.
Fig 1A caption is wrong "201 annotated compounds with proper identification detected using GC-MS are plotted on the Principal Component Analysis (PCA) graph". The compounds aren't plotted. The compounds are the 201-axis, the dimension is then reduced to 2-axis with PCA and the locations of the growth stages are plotted.

RESPONSE: Thank you for the clarification. Your observation is technically correct. We have replaced the word plotted. Now, the sentence reads as follows: '201 annotated compounds with proper identification detected using GC-MS were analyzed using a Principal Component Analysis (PCA) graph.'
With respect to editing the colors. The phosphorus levels and growth stages are color-coded, and the information related to each treatment is explained in the legend. We would rather keep the graphs as they are currently.

L205: "We determined that the plant's developmental stage was responsible for the separation of the compounds in three marked groups" to convince the reader that its not P level you would need to not group by anything and plot all the data with stars/dots/crosses and colors.

All these figures desperately need legends (Fig1, S1, S2) and clearly state if there is a PCA per grouping.

RESPONSE: Thank you. This comment was addressed in the previous answers.

L230: explained the most variance.

RESPONSE: Thank you. Following your suggestion, we have corrected this sentence and now it reads as follows: 'For each of these treatments, we found the five top compounds that explained the largest proportion of the variance in the principal components (Table 1).'

L231: 'correlation' isn't really defined here. Its more that the compounds which point in the most similar direction to the two principle components, I would get rid of the bit after the comment.

Change this sentence to: For each of these treatments, we found the five top compounds that explained the largest proportion of the variance.

RESPONSE: Thanks. We added this suggestion in the previous response.
Figs S3-S4 are really intresting! Great results
Same goes for Fig 2 and 3A

RESPONSE: Thank you.

| | |
|---|---|
| | L254: does the 1.75 7 and 28 mM mean that the sum of the concentrations or each have that concentration? Fig 3 caption makes this clear but also make it clear in the manuscript.<br><br>RESPONSE: Good observation. Each compound added to the pool had 1.75 mM, 7 mM and 28 mM concentration. We have added this detail in the manuscript. As explained in the methodology section line 189-195: 'In order to determine the potential cumulative effect of 3-hydroxypropionic acid, malic acid, nicotinic acid, and glutamic acid they were combined and the available phosphorus (mg l-1) was determined by OES-ICP. A phosphorus gradient that included the previous tested concentration (7 mM per compound), a higher (28 mM per compound) and a lower (1.75 mM per compound) concentration was tested in order to compare if the combination of compounds would equal or surpass the effect of a single compound. Each compound added to the pool had 1.75 mM, 7 mM and 28 mM concentration. Thus, the combination effect of four compounds were tested in a liquid NBRIP medium.'<br><br>L255: The 1.75M is really interesting, 7mM of them mixed does worse than 7mM of any individually<br><br>Again, really interesting results with Fig 4, very hard to interpret though there is lots going on.<br><br>RESPONSE: Each compound has a different molarity; therefore we cannot say that the 4 compounds at 1.75 mM add up to 7mM. We conclude that 1) compounds in combination have an additive effect, and that 2) the addition of one does not inhibit the effect of the other.<br><br>L300: …'where optimum conditions are scare' -> where nutrients are scarce ??<br><br>RESPONSE: Thank you. We have corrected this sentence and now it read as follows: 'Thus, we hypothesized that at this growth stage roots did not respond to phosphorus fertilization. A. thaliana is considered a plant that can thrive in marginal soils where optimum nutrient conditions are limited.'<br><br>L304: These papers might be useful for this discussion on maize need for P early on with respect to yield: https://doi.org/10.1007/s11104-011-0814-y. https://www.nrcresearchpress.com/doi/pdfplus/10.4141/P00-093) Take them or leave them<br><br>RESPONSE: Thank you for the recommendation. As suggested, those two relevant papers were included one in the introduction section (Grant et al., 1999) and the second in the discussion section (Nadeem et al., 2011).<br><br>L309: 'similarity' do you mean they grouped close together in the PSA? To talk about similarity you would need to use a norm between the treatments.<br><br>RESPONSE: Thanks for the observation. We are referring to visual similarity. We have corrected by adding: 'In the vegetative stage, the root exudates at 50% and 100% phosphorus showed greater visual similarity in the PCA than the exudates at 25% phosphorus, evidencing an initial sensing from the plant in response to its phosphorus demand.'<br><br>L323: I think your mention of other functions of exudates is a good explanation and something other authors overlook.<br><br>RESPONSE: Thank you.<br><br>346: that 'the' shouldn't be there<br><br>RESPONSE: Thank you; 'the' was removed.<br><br>L351: new paragraph<br><br>RESPONSE: Thank you. |

Powered by Editorial Manager® and ProduXion Manager® from Aries Systems Corporation

| | |
|---|---|
| | L352: mechanism 1. Your results suggest there is no synergy (in fact the opposite) in terms of P solubilisation by root exudates, see my comment about L255, at least for the 4 acids you picked out. But this doesn't disprove your mechanism: I think I read somewhere that certain exudates solubilise P from certain soil surfaces, Al oxides, Fe oxides etc (possibly a paper by Gerke about citrate and malate?) Maybe this is why they exude a mixture of acids? Did the soil-analogue you used have this variability in mineral surfaces which soil has?<br>Nonetheless, I think this deserves some more discussion.<br><br>RESPONSE: We addressed this question in comment L255 above.<br><br>L357. I like point 2, I had never heard of this before.<br><br>RESPONSE: Thank you.<br><br>I don't think Table S1 is mentioned in the manuscript<br><br>RESPONSE: Table S1 show data that is already presented in supplementary figure 3 and 4 in more detail. Therefore, is was deleted it. |
| **Additional Information:** | |
| **Question** | **Response** |
| **Financial Disclosure**<br><br>Enter a financial disclosure statement that describes the sources of funding for the work included in this submission. Review the submission guidelines for detailed requirements. View published research articles from *PLOS ONE* for specific examples.<br><br>This statement is required for submission and **will appear in the published article** if the submission is accepted. Please make sure it is accurate. | J. Y. was supported by National Postdoctoral Program for Innovative Talents (BX201600075) and Natural Science Foundation of Jiangsu Province (BK20170724), Y. H. was supported by China Scholarship Council (No. 201709505007).<br>The funders had no role in study design, data collection and analysis, decision to publish, or preparation of the manuscript. |

Powered by Editorial Manager® and ProduXion Manager® from Aries Systems Corporation

| | |
|---|---|
| **Unfunded studies**<br>Enter: *The author(s) received no specific funding for this work.*<br><br>**Funded studies**<br>Enter a statement with the following details:<br>• Initials of the authors who received each award<br>• Grant numbers awarded to each author<br>• The full name of each funder<br>• URL of each funder website<br>• Did the sponsors or funders play any role in the study design, data collection and analysis, decision to publish, or preparation of the manuscript?<br>• **NO** - Include this sentence at the end of your statement: *The funders had no role in study design, data collection and analysis, decision to publish, or preparation of the manuscript.*<br>• **YES** - Specify the role(s) played.<br><br>* typeset | |
| **Competing Interests**<br><br>Use the instructions below to enter a competing interest statement for this submission. On behalf of all authors, disclose any competing interests that could be perceived to bias this work—acknowledging all financial support and any other relevant financial or non-financial competing interests.<br><br>This statement **will appear in the published article** if the submission is accepted. Please make sure it is accurate. View published research articles from *PLOS ONE* for specific examples. | The authors have declared that no competing interests exist. |

| | |
|---|---|
| **NO authors have competing interests**<br><br>Enter: *The authors have declared that no competing interests exist.*<br><br>**Authors with competing interests**<br><br>Enter competing interest details beginning with this statement:<br><br>*I have read the journal's policy and the authors of this manuscript have the following competing interests: [insert competing interests here]*<br><br>* typeset | |
| **Ethics Statement**<br><br>Enter an ethics statement for this submission. This statement is required if the study involved:<br><br>• Human participants<br>• Human specimens or tissue<br>• Vertebrate animals or cephalopods<br>• Vertebrate embryos or tissues<br>• Field research<br><br>Write "N/A" if the submission does not require an ethics statement.<br><br>General guidance is provided below. Consult the submission guidelines for detailed instructions. **Make sure that all information entered here is included in the Methods section of the manuscript.** | N/A. |

| | |
|---|---|
| **Format for specific study types**<br><br>**Human Subject Research (involving human participants and/or tissue)**<br>• Give the name of the institutional review board or ethics committee that approved the study<br>• Include the approval number and/or a statement indicating approval of this research<br>• Indicate the form of consent obtained (written/oral) or the reason that consent was not obtained (e.g. the data were analyzed anonymously)<br><br>**Animal Research (involving vertebrate animals, embryos or tissues)**<br>• Provide the name of the Institutional Animal Care and Use Committee (IACUC) or other relevant ethics board that reviewed the study protocol, and indicate whether they approved this research or granted a formal waiver of ethical approval<br>• Include an approval number if one was obtained<br>• If the study involved *non-human primates*, add *additional details* about animal welfare and steps taken to ameliorate suffering<br>• If anesthesia, euthanasia, or any kind of animal sacrifice is part of the study, include briefly which substances and/or methods were applied<br><br>**Field Research**<br><br>Include the following details if this study involves the collection of plant, animal, or other materials from a natural setting:<br>• Field permit number<br>• Name of the institution or relevant body that granted permission | |
| **Data Availability**<br><br>Authors are required to make all data underlying the findings described fully available, without restriction, and from the time of publication. PLOS allows rare exceptions to address legal and ethical concerns. See the PLOS Data Policy and FAQ for detailed information. | Yes - all data are fully available without restriction |

*Powered by Editorial Manager® and ProduXion Manager® from Aries Systems Corporation*

| | |
|---|---|
| A Data Availability Statement describing where the data can be found is required at submission. Your answers to this question constitute the Data Availability Statement and **will be published in the article**, if accepted.<br><br>**Important:** Stating 'data available on request from the author' is not sufficient. If your data are only available upon request, select 'No' for the first question and explain your exceptional situation in the text box.<br><br>Do the authors confirm that all data underlying the findings described in their manuscript are fully available without restriction? | |
| **Describe where the data may be found in full sentences. If you are copying our sample text, replace any instances of XXX with the appropriate details.**<br><br>• If the data are **held or will be held in a public repository**, include URLs, accession numbers or DOIs. If this information will only be available after acceptance, indicate this by ticking the box below. For example: *All XXX files are available from the XXX database (accession number(s) XXX, XXX.)*.<br>• If the data are all contained **within the manuscript and/or Supporting Information files**, enter the following: *All relevant data are within the manuscript and its Supporting Information files.*<br>• If neither of these applies but you are able to provide **details of access elsewhere**, with or without limitations, please do so. For example:<br><br>*Data cannot be shared publicly because of [XXX]. Data are available from the XXX Institutional Data Access / Ethics Committee (contact via XXX) for researchers who meet the criteria for access to confidential data.*<br><br>*The data underlying the results presented in the study are available from (include the name of the third party* | All relevant data are within the manuscript and its Supporting Information files. |

| | |
|---|---|
| *and contact information or URL).*<br>• This text is appropriate if the data are owned by a third party and authors do not have permission to share the data.<br><br>* typeset | |
| Additional data availability information: | Tick here if the URLs/accession numbers/DOIs will be available only after acceptance of the manuscript for publication so that we can ensure their inclusion before publication. |

April 06, 2020

Center of Rhizosphere Biology

Colorado State University, Fort Collins, Colorado.

Dear Managing Editor,

We would like to submit our manuscript entitled: "Role of root exudates in assimilation of phosphorus in young and old *Arabidopsis thaliana* plants" for consideration as a *PLOS ONE* research article.

This manuscript provides new perspectives on deciphering plant total exudate changes in response to phosphorus addition. We believe that the findings presented in our paper will appeal to the reader interested in root biology, root exudation, phosphorus fertilization, and general nutrient acquisition by roots.

This manuscript is original work and is not under consideration by any other journal. All authors approved the manuscript and this submission.

Sincerely,

Jorge Vivanco

Corresponding Author

Manuscript

Click here to access/download;Manuscript;Manuscript-PO-0406.docx

# Role of root exudates on assimilation of phosphorus in young and old *Arabidopsis thaliana* plants

Hugo A. Pantigoso [1], Jun Yuan[2], Yanhui He[1,3], Qinggang Guo[1,4], Charlie Vollmer[5], Jorge M. Vivanco[1]*

[1] *Center for Rhizosphere Biology and Department of Horticulture and Landscape Architecture, Colorado State University, Fort Collins, USA*;

[2] *Jiangsu Provincial Key Lab of Organic Solid Waste Utilization, Jiangsu Collaborative Innovation Center for Organic Solid Waste Utilization, Nanjing Agricultural University, Nanjing, China*;

[3]*Key laboratory for Green Processing of Chemical Engineering of Xinjiang Bingtuan, School of Chemistry and Chemical Engineering, Shihezi University, Shihezi, China*;

[4]*Institute of Plant Protection, Hebei Academy of Agricultural and Forestry Science, Baoding, China*;

[5]*Department of Statistics, Colorado State University, Fort Collins, USA*

*Corresponding author:

E-mail: J.Vivanco@colostate.edu

# Abstract

The role of root exudates has long been recognized for its potential to improve nutrient use efficiency in cropping systems. However, studies addressing the variability of ~~roots~~ exudates involved in phosphorus solubilization across plant developmental stages remain scarce. ~~In this study, we tested the effect of three increasing phosphorus fertilization levels on roots' exudate composition of *Arabidopsis thaliana* at three developmental stages (seedling, vegetative and bolting) under sterile nutrient solution-based conditions~~. The ~~data showed that the total~~ exudates changed in response to the incremental addition of phosphorus starting ~~at~~ the vegetative ~~level.~~ It ~~was found that specific~~ metabolites decreased in ~~quantity to phosphorus supplementation~~ at specific stages of development. Some of those metabolites were tested for their ~~phosphorus solubilization~~ activity, and ~~it was found that~~ 3-hydroxypropionic acid, malic acid, and nicotinic acid were able to solubilize ~~phosphorus under solid and liquid NBRIP media conditions.~~ In summary, our data suggest that plants can release distinct compounds to deal with phosphorus deficiency needs influenced by the phosphorus nutritional status at varying developmental stages.

# Introduction

Phosphorus is an essential element for plant growth and development [1], and a non-renewable resource [2,3]. Despite the fact that the total amount of phosphorus is high in most agricultural soils, crop yields are often limited by low availability due to the non-soluble form and low mobility of this nutrient [4,5]. It has been estimated that residual phosphorus fertilizer known as 'phosphorus legacy' in soil can be sufficient to sustain crop yield for the next century and could alleviate expected phosphorus shortages in the next 50 years [6]. Hence, studies addressing potential solutions to exploit soil phosphorus reserves are needed.

Plants have developed several strategies for acquisition of phosphorus in low nutrient environments mainly by modifying root structure and changing the soil chemical properties in the rhizosphere [7]. These mechanisms include longer root formation and increases of root:shoot ratio allowing the transport of phosphorus from the roots to the shoots [8,9]. Certain plants such as *Lupinus* sp. can promote the formation of cluster roots to secrete phosphorus solubilizers such as citrate and malate in sufficient quantities to lower the rhizosphere pH, thus enhancing the movement of phosphorus and consequently plant uptake [10,11]. The secretion of phosphorus solubilizers is not restricted to cluster root-forming plants. Several other species such as alfalfa, spinach and radish have also been documented to increase the efflux of organic anions as a result of a lack of phosphorus available in the soil [12-14].

Increasing phosphorus solubility can also be achieved by modifying the rhizosphere chemistry. Root-secreted phosphorus solubilizers are capable of increasing solubility of a variety of insoluble phosphorus forms in the soil such as organic phosphorus, inorganic phosphorus like calcium phosphate, and humic substances bonded to phosphorus anions such as Al (III) and Fe (III). They can be classified as protons or $OH^-$/$HCO_3^-$ equivalents, redox equivalents and di- and

tricarboxylic acid anions [13]. The mechanisms used by plants to solubilize phosphorous vary according to the plant species, nutritional status of the plant and soil and environmental conditions [15]. However, organic acids such as oxalate, citrate and malate are recurrent in a variety of plant species and thereby are the most studied means used by plants to solubilize phosphorous [13]. Considerably less research has been performed to explore the total root exudate profile and to identify other compounds exerting similar and complementary functions in the rhizosphere~~ of *A. thaliana* across its phenological stages~~.

All plants share a similar need for phosphorus, but this need differs broadly based on the crop type and its developmental stage [16,17]. In general, most crops require early phosphorus supplementation for optimum yield [18]. Nevertheless, higher amounts of phosphorus in later growth stages are required proportional to increases in the biomass of the plant [19-22]. Due to the variation of phosphorus demand during the plant's lifetime, it becomes necessary to fully understand fluctuations of root exudates as a means to solubilize the phosphorus present in the ~~P legacy of~~ the soil.

In this study, we tested the effect of three ~~phosphorus~~ fertilization levels on root exudates~~'~~ composition of *A. thaliana* at distinct developmental stages. We hypothesized that ~~phosphorus~~ amendment would negate the intrinsic ability of certain root exudate compounds to solubilize inorganic phosphorus. The results showed that the total exudates changed in response to the addition of ~~phosphorus,~~ and that certain metabolites were reduced under increasing ~~phosphorus~~ amendments at varying growth points. As a proof of concept, some of the metabolites that decreased in quantity to the phosphorus addition were tested and four of them were found to solubilize ~~phosphorus.~~

# Materials and methods

## Plant growth conditions, phosphorus fertilization and collection of root exudates

A. *thaliana* (L.) Heynh wild-type Columbia seeds were obtained from Lehle Seeds (Round Rock, USA) and surface sterilized with Clorox bleach (Sodium Hypochlorite, 8.25%) for 1 min. Seeds were rinsed with distilled water 3 times and plated on different phosphorus levels of Murashige and Skoog (MS) agar (1.5%) (supplemented with 3% sucrose) in square plates. The plates were placed vertically in a growth chamber (Percival Scientific) at 25 ± 2 °C with a photoperiod of 16 h : 8 h, light : dark for germination. The germinated seedlings were grown in three phosphorus levels: full strength (100%, 1.25mM), half (50%, 0.625mM) and a quarter (25%, 0.3125mM) in solid MS medium as described above. The three phosphorus levels used in this study did not stimulate the plant starvation response, which is generally activated at values below 2 µM of phosphorus in the soil solution [23]. Instead, we followed a low and high fertilization regime used commercially in agriculture [24]. phosphorus concentration was adjusted with $Na_2HPO_4$ and $NaH_2PO_4$, and phosphorus -free MS medium was used as basal medium. After seven days of growth, the seedlings were transferred to six well plates, each well containing 5 ml of liquid MS with 1% sucrose with one individual seedling per well containing distinct phosphorus levels as described above.  The seedlings grown in solid MS at the particular phosphorus level were placed under the same phosphorus level under liquid M conditions. All the plates were placed on an orbital shaker at 90 rpm under 25 ± 2 °C and lighted by cool white fluorescent light (45µmol $m^{-2}$ $s^{-1}$) with a photoperiod of 16 h : 8 h, light : dark. The nutrient solutions were replaced every week by transferring the plants to new six well plates with 5 ml of fresh liquid MS medium under the same phosphorus levels as stated above. Root exudates of different developmental stages were collected as follows: 1-3 days after transplanting for

seedling stage, 8-10 days after transplanting for vegetative stage, and 15-17 days after transplanting for bolting stage. Prior to the collection of root exudates, plants were removed and washed mildly with sterile water and subsequently placed in new wells containing 5 ml of sterile water for two days. The solution in which the plants were floating was collected and considered as the root exudates. The solution of one plate containing six wells with six individual plants was pooled and considered as one replicate. We used three replicates for each stage under three phosphorus levels. The root exudates were filtered through a 0.45 µm (Millipore, MA) to remove root sheathing and root border-like cells and were stored at -20 °C for Gas Chromatography-Mass Spectrometry (GC-MS) analysis.

## Gas Chromatography-Mass Spectrometry (GC-MS) of root exudates

To characterize the chemical composition, root exudates that were freeze-dried were then processed as described by Chaparro et al, [25] and subjected to Gas Chromatography-Mass Spectrometry (GC-MS) analyses at the Genome Center Core Services, University of California Davis. Extracts were briefly dried under nitrogen gas and then methoximated and trimethylsilylated. The derivatives were analyzed by an Agilent 6890 gas chromatograph (Santa Clara, CA) containing a 30-m-long, 0.25-mm inner diameter rtx5Sil-MS column with an additional 10 m integrated guard column. Metabolites were detected using the BinBase algorithm [26] and identified by comparing the retention index and mass spectrum of each analyte against the Fiehn mass spectral library from the West Coast Metabolomics Center, University of California Davis.

## Statistical analysis of total root exudate data

To discover the differential expression levels of compounds across the plant's growth stages and fertilizer levels, R statistical software (R Core Team, 2017) was used to perform Principal Components Analysis (PCA) on all annotated compounds. For each of the following analyses, we performed a PCA by first separating the total root exudate data by plant growth stage. Within each plant growth stage (e.g. Bolting), we performed a centered and scaled PCA. By verifying that the first two principal components explained a sufficient amount of the variance in the compound(s) expression levels, we were able to determine which compounds had the highest correlation with these components through the magnitude of the variance. The variances are representative of a compound at each growth stage and at a particular phosphorus level. The largest variance represents the highest correlation to the principal components. This method allowed us to determine which compounds explained most of the variance across the fertilizer levels and for each of the plant's growth stages.

Significant differences between phosphorus amendment and compound counts level per developmental stage were analyzed using a one-way ANOVA. Tukey HSD test was used to identify significance ($p < 0.05$) among phosphorus treatments.

## Qualitative analysis of phosphorus solubilizing ability of compounds derived from root exudates

From a list of selected compounds only 13 of them diluted in dd$H_2O$ at the desired concentration (100 mM) (Table 1). We used the reported concentration of organic acids in the rhizosphere (1 μM to 100 mM) as reference to select the concentration of the compounds tested in this study [27-29]. These 13 compounds were qualitatively evaluated for their phosphorus

solubilizing abilities on NBRIP (National Botanical Research Institute phosphate growth medium) solid medium containing glucose 10.0 g, $Ca_3(PO_4)_2$ 5.0 g, NaCl 0.2 g, $MgSO_4 \cdot 7H_2O$ 0.5 g, $(NH_4)_2SO_4$ 0.5 g, KCl 0.2 g, $MnSO_4$ 0.03 g, $FeSO_4 \cdot 7H_2O$ 0.003 g, agar 12 g in 1 L water, pH：7.0-8.0 [30]. All compounds used in this experiment were purchased from Thermo-Fisher Scientific. The specific solution (100 μL) of each compound was placed on NBRIP solid medium. The plates were inoculated at room temperature and let to sit overnight. phosphorus solubilizing ability was visually judged as a clear halo around every drop of solution containing the given compound.

**Table 1**. Five top compounds from root exudates identified by its spatial separation on the Principal Component Analysis (PCA). Compounds are divided by phosphorus level and plant developmental stages. (*) are compounds diluted in ddH2O at 100 mM.

| Growth Stage | P level (%) | | Compound name | |
|---|---|---|---|---|
| Seedling | N/A | | N/A | |
| Vegetative | 25 | 1 | Nicotinic acid | * |
| | | 2 | 4-hydroxybutyric acid | * |
| | | 3 | 3-hydroxypropionic acid | * |
| | | 4 | 1-monostearin | |
| | | 5 | 1-monopalmitin | |
| Vegetative | 50 and 100 | 6 | threonine | * |
| | | 7 | proline | * |
| | | 8 | O-acetylserine | * |
| | | 9 | Leucine | * |
| | | 10 | Alanine | * |
| Bolting | 25 | 11 | Threonic acid | |
| | | 12 | Octadecanol | |
| | | 13 | Malic acid | * |
| | | 14 | Glycine | * |
| | | 15 | Galactinol | * |
| Bolting | 50 | 16 | scopeletin | |
| | | 17 | phenylacetamine | |
| | | 18 | 5-aminovaleric acid | * |
| | | 19 | 1-monopalmitin | |
| | | 20 | 1-monoheptadecanoyl glyceride NISTT | |
| Bolting | 100 | 21 | Sophorose | |
| | | 22 | Guanine | |
| | | 23 | Glutamic acid | * |
| | | 24 | Adenine | |
| | | 25 | 1-Kestose | |

## Quantitative analysis of phosphorus solubilizing ability of water-soluble compounds (individually and combined)

For quantification ~~analysis,~~ 35 µL of the same concentration previously tested (100 mM) of the 13 compounds were added to 5 mL liquid NBRIP medium resulting in a final concentration of 7mM. The tubes were then placed at a continuous agitation at 150 rpm on a rotary shaker for 72 h. Afterwards, the solution was centrifuged at 6000 rpm for 5 min, and the supernatant was filtered with 0.2 µm filter (Thermo-Fisher Scientific). Liquid NBRIP medium without compound addition was used as control. The concentration of ~~soluble~~ phosphorus in the supernatant was analyzed accordin~~gly with~~ the protocol ~~by~~ Soltanpour et al, [31] and measured by Inductive Coupled Plasma-Optical Emission Spectrometer (ICP-OES) (Perkin Elmer 7300DV) at the Soil, Water and Plant Testing Laboratory of Colorado State University. This experiment and analysis were repeated twice with 3 repetitions.

In order to determine the potential cumulative effect of 3-hydroxypropionic acid, malic acid, nicotinic acid, and glutamic acid they were combined and the available phosphorus (mg $l^{-1}$) was determined by OES-ICP. A phosphorus ~~gradient~~ that included the previous tested concentration (7 mM per compound), a higher (28 mM per compound) and a lower (1.75 mM per compound) concentration was tested in order to compare if the combination of compounds would equal or surpass the effect of a single compound. Each compound added to the pool had 1.75 mM, 7 mM and 28 mM concentration. Thus, the combination effect of four compounds were tested in a liquid NBRIP medium.

## Statistical analysis for quantitative phosphate release

Significant differences between phosphorus content measured in NBRIP liquid media was analyzed using a one-way ANOVA. Tukey HSD test was used to identify significant treatments in multiple comparisons. P-value were considered significant below 0.05. Assumptions of normality and homogeneity of the data were confirmed prior to the analysis.

## Results

### Effect of phosphorus levels on *A. thaliana* root exudates at different plant developmental stages

The effect of increasing phosphorus at three different concentrations on the root exudates of *A. thaliana* was analyzed at various developmental stages. In total, 456 compounds were detected by GC-MS among the treatments. The data set was reduced to 201 annotated compounds, and only those with a proper identification were kept for statistical analysis. The grouping of the compounds in the plot maintained the same pattern even after subtracting the non-annotated compounds (S1 Fig), suggesting that all of the differentially expressed compounds were indeed annotated by the GC-MS analyses.

The variability in our data, after subtracting the non-annotated compounds, was analyzed using a principal component analysis (PCA) where variability of component 1 (PC1) accounted for 29.8%, while component 2 (PC2) accounted for 21.7%. We determined that the plant's developmental stage was responsible for the separation of the compounds in three marked groups: seedling, vegetative and bolting (Fig 1A) as previously reported [25,32,33]. When fertilizer levels (25% phosphorus, 50% phosphorus and 100 % phosphorus) were applied to each developmental stage, segregation was observed in certain developmental stages and P levels (S2 Fig). Overall, phosphorus levels did not cause a separation effect on the root exudates patterns of

seedling stage, but the event was observed in the vegetative and particularly in the bolting phases (Fig 1A). In the vegetative stage, a clear parting was observed at 25% phosphorus compared to the 50 and 100% treatments (Fig 1B). In the bolting stage, there was a clear division between the three fertilizer levels, but the 50% level was the most secluded rate (Fig 1C).

## Differences in compound-levels in the vegetative and bolting stages due to phosphorus fertilization

A separate analysis was performed to determine correlations between different and highly expressed compounds for a specific fertilization level and developmental stage. We focused our analysis on treatments 25% phosphorus at the vegetative stage, and 25%, 50% and 100% phosphorus at the bolting stage because these treatments showed to have the highest dissimilarity in the PCA. In addition, treatments 50% and 100% at the vegetative stage were grouped because they were clustered in the PCA. For each of these treatments, we found the five top compounds that explained the largest proportion of the variance in the principal components (Table 1). In addition, the expression levels of the compounds based on phosphorus level and growth stage were determined (S3-S4 Figs). We found some interesting patterns, such as that some compounds decreased expression upon increased phosphorus fertilization (e.g. 3-hydroxypropionic acid, malic acid, galactinol), while other compounds showed a positive correlation with phosphorus amendment (e.g. guanine, glutamic acid, sophorose).

## Root-exudate metabolites solubilize calcium phosphate in solid and liquid media

We then tested the ability of some of the selected compounds irrespective of their expression level upon phosphorus fertilization to induce phosphorus solubilization. We found

that 3-hydroxypropionic acid, malic acid, and nicotinic acid formed a clear halo when tested at a concentration of 100 mM, indicating the ability of this compound to solubilize phosphorus from calcium phosphate (Fig 2). All the remaining tested compounds did not present phosphorus solubilizing ability at 100 mM.

The phosphorus -solubilizing effect of the selected compounds was further tested using a more sensitive technique (OES-ICP) at a final concentration of 7 mM in liquid NBRIP medium. The results showed that in addition to 3-hydroxypropionic acid, malic acid, and nicotinic acid, glutamic acid was also found to express phosphorus solubilizing ability on sterile nutrient solution using spectroscopy measurements. The results showed that the soluble P in the medium without compound addition was 5.34 mg $l^{-1}$ (control) compared to 86.38, 48.61, 48.46, and 53.92 mg $l^{-1}$ when 3-hydroxypropionic acid, malic acid, nicotinic acid, and glutamic acid were added (Fig 3A).

Further analysis aimed to test the combined effect of all the four compounds on NBRIP medium resulting in 34.5 (for 1.75 mM) 101.21 (for 7mM) and 211.63 (for 28 mM) mg $l^{-1}$ of available P compared to 4.69 mg $l^{-1}$ from the control (Fig 3B).

## The plant mediates changes in levels of phosphorus solubilizer compounds in response to phosphorus status and developmental stage

We further analyzed the expression levels upon phosphorus fertilization of 3-hydroxypropionic acid, malic acid, nicotinic acid, and glutamic acid in the different developmental stages. Depending on the compound, our results showed that the cumulative secretion levels of the compounds increased, decreased or remained statistically similar ($p>0.05$)

~~following increasing levels of phosphorus~~ amendment (Fig 4). At the seedling stage, ~~changes in~~ cumulative secretion ~~for~~ the four ~~phosphorus~~ solubilizers ~~were not statistically significant,~~ which agrees with the PCA analysis (Fig 1A). Interestingly, at the vegetative stage nicotinic acid and 3-hydroxypropionic acid showed higher abundance at the lowest phosphorus level (0.3125 mM) and decreased significantly ($p<0.05$) for the two higher ~~P~~ levels (0.625 and 1.25 mM). Malic and glutamic acid followed a similar pattern; however, their changes were not statistically significant. At the bolting stage, differences of compound cumulative secretion were observed for glutamic acid, malic acid and nicotinic acid, but not for 3-hydroxypropionic acid which did not increase or decrease following an incremental phosphorus level. Cumulative secretion of malic acid was reduced significantly from 0.3125 to 0.625 mM ~~phosphorus treatments~~, and then incremented its cumulative secretion for the highest ~~phosphorus~~ treatment (1.25 mM). However, secretion levels of malic acid for the two highest ~~phosphorus~~ treatments (0.625 and 1.25 mM) were below the value for the lowest ~~phosphorus~~ rate (0.3125 mM) (Fig 4A). Similarly, cumulative secretion of nicotinic acid increased significantly from 0.3125 to 0.625 mM but dropped for 1.25 mM of ~~phosphorus~~ (Fig 4C). Lastly, glutamic acid consistently increased upon higher ~~phosphorus~~ fertilization reaching its peak at 1.25 mM ~~of phosphorus~~ (Fig 4B).

# Discussion

For the most part, the influence of phosphorus fertilization on root secretion has been studied at specific developmental stages [34,35], among genotypes of the same plant species [36] or between different species [37]. For instance, Vengavasi et al. [36] found cultivar-dependent differences in root exudation of soybeans grown under phosphorus -sufficient versus phosphorus -deficient conditions. It is worth noting that these plants were sampled at the reproductive stage

of growth, a metabolically active stage with higher demand for energy and phosphorus nutrition. In a different study, the authors found differences in root exudation in maize seedlings grown in phosphorus -sufficient and phosphorus -deficient conditions [34]. In contrast, here we studied the interplay of increasing phosphorus fertilization on root exudation at different plant developmental stages (seedling, vegetative and bolting) in *A. thaliana*.

Our results showed that the root exudate profiles were similar within the seedling stage across all phosphorus fertilization treatments (Fig 1A). Thus, we hypothesized that at this growth stage roots did not respond to phosphorus fertilization. *A. thaliana* is considered a plant that can thrive in marginal soils where optimum nutrient conditions are limited [38], and at early developmental stages the plant does not require high amounts of phosphorus as a mechanism to cope with poor soil conditions [39]. In fact, it has been shown that the reserves of phosphorus in the seeds of several plants, including *Brassicaceae* species, can support seedling growth in a medium lacking phosphorus for at least four weeks after germination [40-42,39]. In contrast, maize, a monocotyledonous plant, possesses two genes induced by phosphate starvation in its genomes compared to five in dicotyledonous such as *A. thaliana* [43,44]; suggesting a difference in phosphorus responsiveness between these two plant groups. In addition, signs of phosphorus scarcity in dicotyledonous (e.g. *A. thaliana*) is often observed at later stages of growth [45]. In the vegetative stage, the root exudates at 50% and 100% phosphorus showed greater visual similarity in the PCA than the exudates at 25% phosphorus, evidencing an initial sensing from the plant in response to its phosphorus demand. At the bolting stage, the three amounts of phosphorus fertilization showed distinct root exudation patterns. These results may suggest that as the plant ages the demand for phosphorus increases, as evidenced by the differential root exudation profiles [46]. This result is in accordance with Tawaraya et al. [47], who showed that

phosphorus content increased in shoot and root-dried soybean tissues as the plant developed, and that root exudates' content, collected on day 1, 5, 10 and 15 of growth, increased for phosphorus-depleted compared to phosphorus -sufficient treatments.

Our results suggest that plants in the vegetative stage only sense the 25% phosphorus as low levels, whereas plants at bolting stage sense both 25% and 50% phosphorus as low. This pattern of incremental phosphorus requirement during progression in plant development is common in annual plants such as *A. thaliana* [39]. For instance, *Brassica napus* L. requires an incremental supply of phosphorus at flowering onset, which is critical for protein and oil synthesis, and the development of seeds [48,49]. It is worth noting that the intermediate phosphorus rate (50%) at bolting stage was largely secluded (in the PCA plot) compared to 25% and 100% treatments, indicating a higher dissimilarity in root exudation composition. This could be due to the fact that the functions of plant metabolites are diverse and are not restricted only to nutrient acquisition. For instance, root exudates can be substrates, chemotactic or signaling molecules that regulate plant root and microbial interactions [50]. Such plant modulation can be specific to developmental stages [51].

The PCA data allowed for the visual determination of changes in root exudation composition between plant developmental stages and phosphorus fertilization levels. We then developed a list of compounds based on those differences observed in the PCA in response to phosphorus nutrient status; and four of those compounds (i.e. 3-hydroxypropionic acid, nicotinic acid, glutamic acid and malic acid) were confirmed as phosphorus solubilizers. Three (3-hydroxypropionic acid, nicotinic acid, and malic acid) out of the four compounds were significantly more abundant at the lowest phosphorus rate and reduced in concentration as phosphorus amendments were elevated. Our findings agree with a variety of studies evaluating

the exudation of organic acids (e.g. malic acid) in ~~different~~ plant species under various phosphate availabilities [52,13]. Malic acid is a primary compound released by roots under phosphorus deficiency, but often not the most effective [52]. In contrast, nicotinic acid and glutamic acid are less abundant than malic acid or oxalic acid in plant species [46,53]. 3-Hydroxypropionic acid has not been previously associated with ~~phosphorus~~ solubilization, however it has been described as a natural product of a plant endophytic fungus [54,55]. Further, the ~~understanding~~ of the ~~dynamic~~ of these compounds throughout plant phenology is scarce mainly because these studies are often performed in hydroponic systems limiting the root exudate collection to early stages of plant development for a variety of plant crops [52,56]. In our study, we observed that the cumulative secretion of malic acid significantly increased during low ~~phosphorus~~ availability, but it was ~~restricted to~~ the bolting stage. 3-Hydroypropionic acid and nicotinic acid followed the same pattern, however they were significantly ~~expressed~~ only the in vegetative stage. Conversely, glutamic acid and nicotinic acid increased in abundance at bolting stage when ~~phosphorus~~ levels increased. Interestingly, nicotinic acid switched cumulative secretion depending on the developmental stage. It has been reported that nicotinic acid induced flowering in *Lemna* plants [57], and that nicotinic acid can alleviate abiotic plant stresses by increasing ~~phytohormones~~ levels such as indol-3-acetic acid ~~(IAA)~~ and gibberellic acid ~~($GA_3$)~~ [58].

Based on these observations we hypothesize that plant developmental stage modulates root exudation to deal with phosphorus deficiency by three potential mechanisms: (1) Plants secrete synergistic ~~phosphorus~~-solubilizing compounds in stages of high ~~P~~ demand. In this study nicotinic acid, a moderate ~~phosphorus~~ -solubilizer, was released in combination with 3-hydroxypropionic acid, a stronger phosphorus-solubilizer, ~~improving phosphorus acquisition at the vegetative stage (see Fig 3)~~. This agrees with a recent study showing synergistic association

of citrate and phytase to improve acquisition of plant unavailable phosphorus in tobacco in the vegetative stage [59]. (2) Plants prevent the degradation of ~~phosphorus-solubilizer~~ compounds such as malic acid, rapidly degraded by soil microbes [60], by releasing a different compound such as 3-hydroxypropionic acid preceding a growth stage of high phosphorus demand. It has been shown that certain plants, such as lupin, can release compounds that inhibit microbial activity to reduce organic acid degradation prior to the release of organic acids. [61]. Lastly, (3) plants secrete specific compounds to mediate either direct nutrient solubilization or the proliferation of distinct microbial taxa (with ~~phosphorus~~ solubilizing activity) at specific growth stages (e.g. vegetative, bolting). In support of this hypothesis, root exudates can promote the activity of symbiotic microbes, such as ~~phosphorus~~ solubilizing bacteria and siderophore-releasing bacteria and exert mobilization of non-available plant nutrients in soils at a single growth stage [62-64].

It has been estimated that organic acids constitute 5 to 10 % of the total organic carbon in the soil solution. The concentration of organic anions measured in the soil solution usually ~~range~~ from 100 nM to more than 580 ~~u~~M in the rhizosphere of cluster roots [65]. However, millimolar concentrations of organic anions are likely required in the soil solution to effectively increase soluble ~~P~~ concentration especially in calcareous soils [66, 67]. Strom et al. [66] tested three organic acids (citrate, malic and oxalate) and a wide range of concentrations (1 mM to 100 mM) to evaluate its effects on the mobilization of phosphorus in calcareous soil. The results showed that the phosphorus mobilization of the tested compounds had a low efficiency and its effect varied depending on the type of organic acid, compound concentration, and pH. Further, due to the low phosphorus mobilization efficiency of those compounds it is still argued if the benefit of releasing large amounts of organic acids into the soil will exceed the cost of carbon lost by the

plant, which can be seen as an unnecessary trade-off [66]. However, low efficiency organic acids can be particularly important in phosphorus mobilization for calcareous soils with a limited phosphorus availability for plants. Finally, our evidence supports the above-mentioned hypothesis, that plants release a combination of compounds with different phosphorus-solubilizing efficiencies, at specific stages of growth, to deal with particular phosphorus needs.

Lastly, root exudates from liquid culture systems allow the determination of exudation rates unaltered by the soil matrix or microbial decomposition if performed under sterile conditions as we did in this study [56]. However, the quality and quantity of the root exudate profile may be impacted by the nutrient solution culture method (also known as hydroponic methods) [68]. Soil-hydroponic hybrids methods for root exudation collection are not exempt of potential physical/physiological perturbances. Thus, sterile nutrient solution culture methods remain especially important to assess temporal dynamics of root exudates.

In summary, the significance of these findings relies on the potential of customizing specific metabolites to be utilized as soil amendments under the most limiting phosphorus conditions and most demanding stage of plant growth. The role of secondary metabolites in phytoremediation efforts has been previously investigated [69] as well as the use of customized synthetic bacteria communities to modify plant phosphate accumulation [70,71]. However, the use of customized metabolites for phosphorus acquisition remains unexplored.

# Conclusions

The data collected indicate that root exudate patterns change as a response to the supply levels of phosphorus, and this change was accentuated as the plant reached maturity, when phosphorus demands are higher. 3-Hydroxypropionic acid (3-HA) and nicotinic acid

accumulated significantly at the vegetative stage under lower phosphorus supplementation and was found to solubilize phosphorus under both solid and liquid medium. This study sheds light on the influence of plant nutrient status and plant phenological growth stages driving the composition of plant root exudates. Future research should focus on understanding the effects of metabolites at a particular developmental stage of growth under phosphorus depleted soil conditions, as well as to test the potential of these phosphorus solubilizer compounds in making phosphorus available for plants grown in soils saturated in unavailable phosphorus forms.

# Conflict of interest

The authors declare no competing interests.

# Acknowledgment

We thank Dr. James Self, manager of the Soil, Water and Plant testing laboratory at Colorado State University for their invaluable advice regarding phosphorous chemical analysis. J. Y. was supported by National Postdoctoral Program for Innovative Talents (BX201600075) and Natural Science Foundation of Jiangsu Province (BK20170724), Y. H. was supported by China Scholarship Council (No. 201709505007).

# References

1. Lynch JP. Roots of the second green revolution. Australian Journal of Botany. 2007 Sep 7;55(5):493-512.
2. Cordell D, Drangert JO, White S. The story of phosphorus: global food security and food for thought. Global environmental change. 2009 May 1;19(2):292-305.
3. Gilbert N. The disappearing nutrient: phosphate-based fertilizers have helped spur agricultural gains in the past century, but the world may soon run out of them. Natasha Gilbert investigates the potential phosphate crisis. Nature. 2009 Oct 8;461(7265):716-9.
4. Torri SI, Correa RS, Renella G. Biosolid application to agricultural land—A contribution to global phosphorus recycle: A review. Pedosphere. 2017 Feb 1;27(1):1-6.
5. Wei Y, Zhao Y, Wang H, Lu Q, Cao Z, Cui H, Zhu L, Wei Z. An optimized regulating method for composting phosphorus fractions transformation based on biochar addition and phosphate-solubilizing bacteria inoculation. Bioresource technology. 2016 Dec 1;221:139-46.
6. Zhu J, Li M, Whelan M. Phosphorus activators contribute to legacy phosphorus availability in agricultural soils: A review. Science of the Total Environment. 2018 Jan 15;612:522-37.
7. Neumann G, Romheld V. The release of root exudates as affected by the plant's physiological status. InThe rhizosphere 2000 Nov 17 (pp. 57-110). CRC press.
8. Bates TR, Lynch JP. Root hairs confer a competitive advantage under low phosphorus availability. Plant and Soil. 2001 Oct 1;236(2):243-50.
9. Heydari MM, Brook RM, Jones DL. The role of phosphorus sources on root diameter, root length and root dry matter of barley (Hordeum vulgare L.). Journal of plant nutrition. 2019 Jan 2;42(1):1-5.

10. Braum SM, Helmke PA. White lupin utilizes soil phosphorus that is unavailable to soybean. Plant and soil. 1995 Sep 1;176(1):95-100.

11. Hocking PJ, Jeffery S. Cluster-root production and organic anion exudation in a group of old-world lupins and a new-world lupin. Plant and Soil. 2004 Jan 1;258(1):135-50.

12. Lipton DS, Blanchar RW, Blevins DG. Citrate, malate, and succinate concentration in exudates from P-sufficient and P-stressed Medicago sativa L. seedlings. Plant Physiology. 1987 Oct 1;85(2):315-7.

13. Gerke J. The acquisition of phosphate by higher plants: effect of carboxylate release by the roots. A critical review. Journal of Plant Nutrition and Soil Science. 2015 Jun;178(3):351-64.

14. Zhang FS, Ma J, Cao YP. Phosphorus deficiency enhances root exudation of low-molecular weight organic acids and utilization of sparingly soluble inorganic phosphates by radish (Raghanus satiuvs L.) and rape (Brassica napus L.) plants. Plant and Soil. 1997 Oct 1;196(2):261-4.

15. Hinsinger P. Bioavailability of soil inorganic P in the rhizosphere as affected by root-induced chemical changes: a review. Plant and soil. 2001 Dec 1;237(2):173-95.

16. Marschner H. Soil- Root Interface: Biological and Biochemical Processes. Soil chemistry and ecosystem health. 1998 Jan 1;52:191-231.

17. Marschner H., 1995. Nutrient availability in soils. In: Marschner, editors. Mineral Nutrition of Higher Plants. London, UK: Academic Press. p. 483-507.

18. Grant CA, Flaten DN, Tomasiewicz DJ, Sheppard SC. The importance of early season phosphorus nutrition. Canadian Journal of Plant Science. 2001 Apr 1;81(2):211-24.

19. Marti HR, Mills HA. Nutrient uptake and yield of sweet pepper as affected by stage of development and N form. Journal of plant nutrition. 1991 Nov 1;14(11):1165-75.

20. Marti HR, Mills HA. Calcium uptake and concentration in bell pepper plants as influenced by nitrogen form and stages of development. Journal of plant nutrition. 1991 Nov 1;14(11):1177-85.

21. Awonaike KO, Kumarasinghe KS, Danso SK. Nitrogen assimilation and distribution in field-grown cowpea at various growth stages. Soil Science Society of America Journal. 1991;55(1):81-5.

22. White PJ, Veneklaas EJ. Nature and nurture: the importance of seed phosphorus content. Plant and soil. 2012 Aug 1;357(1-2):1-8.

23. Föhse D, Claassen N, Jungk A. Phosphorus efficiency of plants. Plant and soil. 1988 Aug 1;110(1):101-9.

24. Sattari SZ, Bouwman AF, Giller KE, van Ittersum MK. Residual soil phosphorus as the missing piece in the global phosphorus crisis puzzle. Proceedings of the National Academy of Sciences. 2012 Apr 17;109(16):6348-53.

25. Chaparro JM, Badri DV, Bakker MG, Sugiyama A, Manter DK, Vivanco JM. Root exudation of phytochemicals in Arabidopsis follows specific patterns that are developmentally programmed and correlate with soil microbial functions. PloS one. 2013;8(2).

26. Fiehn O, Wohlgemuth G, Scholz M, Kind T, Lee DY, Lu Y, Moon S, Nikolau B. Quality control for plant metabolomics: reporting MSI- compliant studies. The Plant Journal. 2008 Feb;53(4):691-704.

27. Jones DL. Organic acids in the rhizosphere–a critical review. Plant and soil. 1998 Aug 1;205(1):25-44.

28. Veneklaas EJ, Stevens J, Cawthray GR, Turner S, Grigg AM, Lambers H. Chickpea and white lupin rhizosphere carboxylates vary with soil properties and enhance phosphorus uptake. Plant and Soil. 2003 Jan 1;248(1-2):187-97.

29. Ström L, Owen AG, Godbold DL, Jones DL. Organic acid behaviour in a calcareous soil implications for rhizosphere nutrient cycling. Soil Biology and Biochemistry. 2005 Nov 1;37(11):2046-54.

30. Nautiyal CS. An efficient microbiological growth medium for screening phosphate solubilizing microorganisms. FEMS microbiology Letters. 1999 Jan 1;170(1):265-70.

31. Soltanpour PN, Jones Jr JB, Workman SM. Optical emission spectrometry. Methods of Soil Analysis: Part 2 Chemical and Microbiological Properties. 1983 Feb 1;9:29-65.

32. Chaparro JM, Badri DV, Vivanco JM. Rhizosphere microbiome assemblage is affected by plant development. The ISME journal. 2014 Apr;8(4):790-803.

33. Yuan J, Chaparro JM, Manter DK, Zhang R, Vivanco JM, Shen Q. Roots from distinct plant developmental stages are capable of rapidly selecting their own microbiome without the influence of environmental and soil edaphic factors. Soil Biology and Biochemistry. 2015 Oct 1;89:206-9.

34. Carvalhais LC, Dennis PG, Fedoseyenko D, Hajirezaei MR, Borriss R, von Wirén N. Root exudation of sugars, amino acids, and organic acids by maize as affected by nitrogen, phosphorus, potassium, and iron deficiency. Journal of Plant Nutrition and Soil Science. 2011 Feb;174(1):3-11.

35. Keerthisinghe G, Hocking PJ, Ryan PR, Delhaize E. Effect of phosphorus supply on the formation and function of proteoid roots of white lupin (Lupinus albus L.). Plant, Cell & Environment. 1998 May;21(5):467-78.

36. Vengavasi K, Pandey R. Root exudation potential in contrasting soybean genotypes in response to low soil phosphorus availability is determined by photo-biochemical processes. Plant Physiology and Biochemistry. 2018 Mar 1;124:1-9.
37. Li L, Li SM, Sun JH, Zhou LL, Bao XG, Zhang HG, Zhang FS. Diversity enhances agricultural productivity via rhizosphere phosphorus facilitation on phosphorus-deficient soils. Proceedings of the National Academy of Sciences. 2007 Jul 3;104(27):11192-6.
38. Mitchell-Olds T, Schmitt J. Genetic mechanisms and evolutionary significance of natural variation in Arabidopsis. Nature. 2006 Jun;441(7096):947-52.
39. White PJ, Veneklaas EJ. Nature and nurture: the importance of seed phosphorus content. Plant and soil. 2012 Aug 1;357(1-2):1-8.
40. Nadeem M, Mollier A, Morel C, Vives A, Prud'homme L, Pellerin S. Relative contribution of seed phosphorus reserves and exogenous phosphorus uptake to maize (Zea mays L.) nutrition during early growth stages. Plant and Soil. 2011 Sep 1;346(1-2):231-44.
41. Nadeem M, Mollier A, Morel C, Vives A, Prud'homme L, Pellerin S. Maize (Zea mays L.) endogenous seed phosphorus remobilization is not influenced by exogenous phosphorus availability during germination and early growth stages. Plant and soil. 2012 Aug 1;357(1-2):13-24.
42. Bolland MD, Baker MJ. High phosphorus concentrations in seed of wheat and annual medic are related to higher rates of dry matter production of seedlings and plants. Australian Journal of Experimental Agriculture. 1988;28(6):765-70.
43. Yang H, Knapp J, Koirala P, Rajagopal D, Peer WA, Silbart LK, Murphy A, Gaxiola RA. Enhanced phosphorus nutrition in monocots and dicots over- expressing a phosphorus-responsive type I H+- pyrophosphatase. Plant biotechnology journal. 2007 Nov;5(6):735-45.

44. Hasan MM, Hasan MM, da Silva JA, Li X. Regulation of phosphorus uptake and utilization: transitioning from current knowledge to practical strategies. Cellular & molecular biology letters. 2016 Dec;21(1):7.
45. Johnson JF, Vance CP, Allan DL. Phosphorus deficiency in Lupinus albus (altered lateral root development and enhanced expression of phosphoenolpyruvate carboxylase). Plant physiology. 1996 Sep 1;112(1):31-41.
46. Neumann G, Massonneau A, Martinoia E, Römheld V. Physiological adaptations to phosphorus deficiency during proteoid root development in white lupin. Planta. 1999 May 1;208(3):373-82.
47. Tawaraya K, Horie R, Shinano T, Wagatsuma T, Saito K, Oikawa A. Metabolite profiling of soybean root exudates under phosphorus deficiency. Soil science and plant nutrition. 2014 Sep 3;60(5):679-94.
48. Lickfett T, Matthäus B, Velasco L, Möllers C. Seed yield, oil and phytate concentration in the seeds of two oilseed rape cultivars as affected by different phosphorus supply. European Journal of Agronomy. 1999 Nov 1;11(3-4):293-9.
49. Rose TJ, Rengel Z, Ma Q, Bowden JW. Post-flowering supply of P, but not K, is required for maximum canola seed yields. European journal of agronomy. 2008 Apr 1;28(3):371-9.
50. Venturi V, Keel C. Signaling in the rhizosphere. Trends in plant science. 2016 Mar 1;21(3):187-98.
51. He Y, Pantigoso HA, Wu Z, Vivanco JM. Co- inoculation of Bacillus sp. and Pseudomonas putida at different development stages acts as a biostimulant to promote growth, yield and nutrient uptake of tomato. Journal of applied microbiology. 2019 Jul;127(1):196-207.

52. Wang Y, Lambers H. Root-released organic anions in response to low phosphorus availability: recent progress, challenges and future perspectives. Plant and Soil. 2019 Feb 12:1-22.

53. Tawaraya K, Horie R, Saito A, Shinano T, Wagatsuma T, Saito K, Oikawa A. Metabolite profiling of shoot extracts, root extracts, and root exudates of rice plant under phosphorus deficiency. Journal of plant nutrition. 2013 Jan 1;36(7):1138-59.

54. Schwarz M, Köpcke B, Weber RW, Sterner O, Anke H. 3-Hydroxypropionic acid as a nematicidal principle in endophytic fungi. Phytochemistry. 2004 Aug 1;65(15):2239-45.

55. Gunatilaka AL. Natural products from plant-associated microorganisms: distribution, structural diversity, bioactivity, and implications of their occurrence. Journal of natural products. 2006 Mar 24;69(3):509-26.

56. Oburger E, Jones DL. Sampling root exudates–mission impossible?. Rhizosphere. 2018 Jun 1;6:116-33.

57. Fujioka S, Yamaguchi I, Murofushi N, Takahashi N, Kaihara S, Takimoto A, Cleland CF. The influence of nicotinic acid and plant hormones on flowering in Lemna. Plant and cell physiology. 1986 Jan 1;27(1):109-16.

58. El-Bassiouny HM. Physiological responses of wheat to salinity alleviation by nicotinamide and tryptophan. International Journal of Agriculture and Biology. 2005;7(4):653-9.

59. Giles CD, George TS, Brown LK, Mezeli MM, Richardson AE, Shand CA, Wendler R, Darch T, Menezes-Blackburn D, Cooper P, Stutter MI. Does the combination of citrate and phytase exudation in Nicotiana tabacum promote the acquisition of endogenous soil organic phosphorus?. Plant and Soil. 2017 Mar 1;412(1-2):43-59.

60. Jones DL. Organic acids in the rhizosphere–a critical review. Plant and soil. 1998 Aug 1;205(1):25-44.

61. Weisskopf L, ABOU- MANSOUR EL, Fromin N, Tomasi N, Santelia D, Edelkott I, Neumann G, Aragno M, Tabacchi R, Martinoia E. White lupin has developed a complex strategy to limit microbial degradation of secreted citrate required for phosphate acquisition. Plant, Cell & Environment. 2006 May;29(5):919-27.

62. O'Banion BS, O'Neal L, Alexandre G, Lebeis SL. Bridging the gap between single-strain and community-level plant-microbe chemical interactions. Molecular Plant-Microbe Interactions. 2020 Feb 15;33(2):124-34.

63. Rolfe SA, Griffiths J, Ton J. Crying out for help with root exudates: adaptive mechanisms by which stressed plants assemble health-promoting soil microbiomes. Current opinion in microbiology. 2019 Jun 1;49:73-82.

64. Voges MJ, Bai Y, Schulze-Lefert P, Sattely ES. Plant-derived coumarins shape the composition of an Arabidopsis synthetic root microbiome. Proceedings of the National Academy of Sciences. 2019 Jun 18;116(25):12558-65.

65. Jones DL. Organic acids in the rhizosphere–a critical review. Plant and soil. 1998 Aug 1;205(1):25-44.

66. Ström L, Owen AG, Godbold DL, Jones DL. Organic acid behaviour in a calcareous soil implications for rhizosphere nutrient cycling. Soil Biology and Biochemistry. 2005 Nov 1;37(11):2046-54.

67. Ryan PR, Delhaize E, Jones DL. Function and mechanism of organic anion exudation from plant roots. Annual review of plant biology. 2001 Jun;52(1):527-60.

68. Oburger E, Dell‘mour M, Hann S, Wieshammer G, Puschenreiter M, Wenzel WW. Evaluation of a novel tool for sampling root exudates from soil-grown plants compared to conventional techniques. Environmental and experimental botany. 2013 Mar 1;87:235-47.
69. Singer AC, Crowley DE, Thompson IP. Secondary plant metabolites in phytoremediation and biotransformation. TRENDS in Biotechnology. 2003 Mar 1;21(3):123-30.
70. Paredes SH, Gao T, Law TF, Finkel OM, Mucyn T, Teixeira PJ, Gonzalez IS, Feltcher ME, Powers MJ, Shank EA, Jones CD. Design of synthetic bacterial communities for predictable plant phenotypes. PLoS biology. 2018 Feb;16(2).
71. Awasthi A. Field-Specific Microbial Consortia Are Feasible: A Response to Kaminsky et al. Trends in biotechnology. 2019 Jun 1;37(6):569-72.

Figure Click here to access/download;Figure;Figure-PO-0406.docx

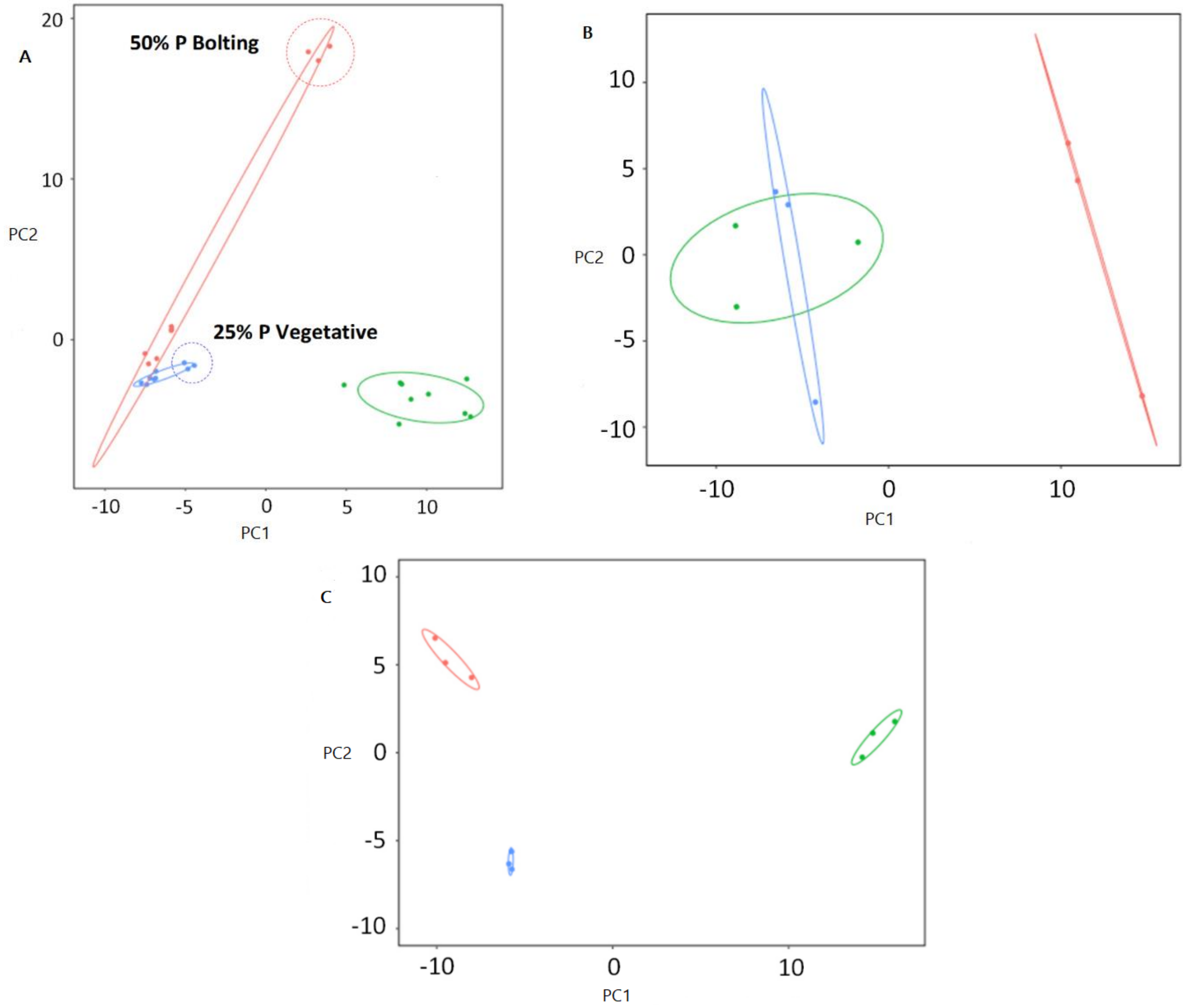


**Fig. 1** Root exudate compounds diverge in response to plant developmental stage and fertilization rate. **(A)** 201 annotated compounds with proper identification detected using GC-MS was analyzed using a Principal Component Analysis (PCA) graph. PCA show dissimilarity of metabolite expression profiles between plant growth stages where PC1 explained 29.8% and PC2 21.7% of the variability. All phosphorus levels (25%, 50% and 100%) are present in each of the plant growth stages shown; seedling (green), vegetative (blue), and bolting (red). Dotted circle highlight clusters of particular fertilization levels. **(B)** Plot of PCA for vegetative stage only where PC1 explained 43.6% and PC2 14.6% of the variability. Compounds grouped by phosphorus treatments: 25% P (red) fertilization clusters separates from 50% P (blue) and 100% P (green). **(C)** Plot of PCA for bolting stage only where PC1 explained 64.3% and PC2 12.9% of

the variability. Compounds grouped by phosphorus treatments: 50% phosphorus (green) separated from 25 % phosphorus (red) and 100% phosphorus.

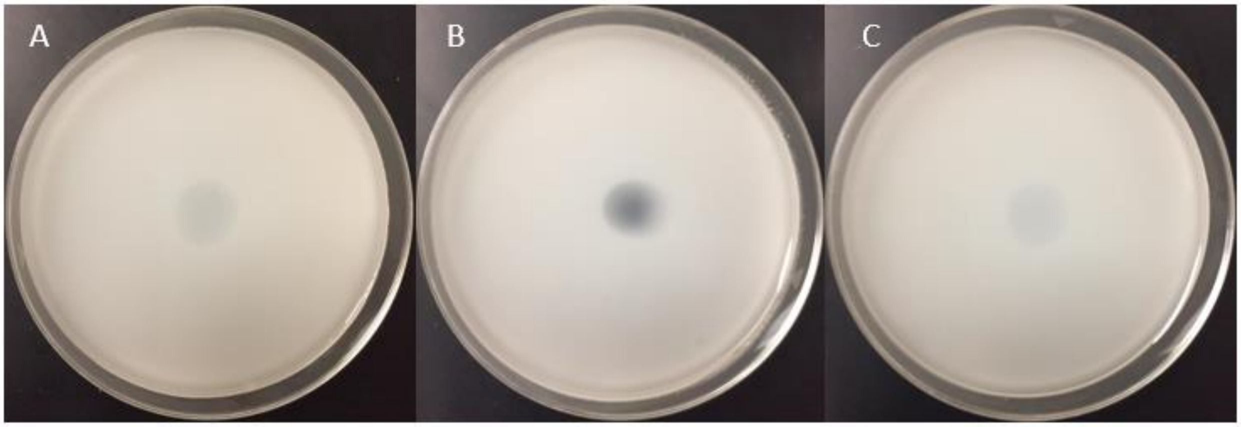


**Fig. 2** Qualitative phosphorus-solubilization analysis of compounds using a calcium-phosphate based medium (NBRIP). **(A)** 3-Hydroxypropionic acid **(B)** malic acid and **(C)** nicotinic acid.

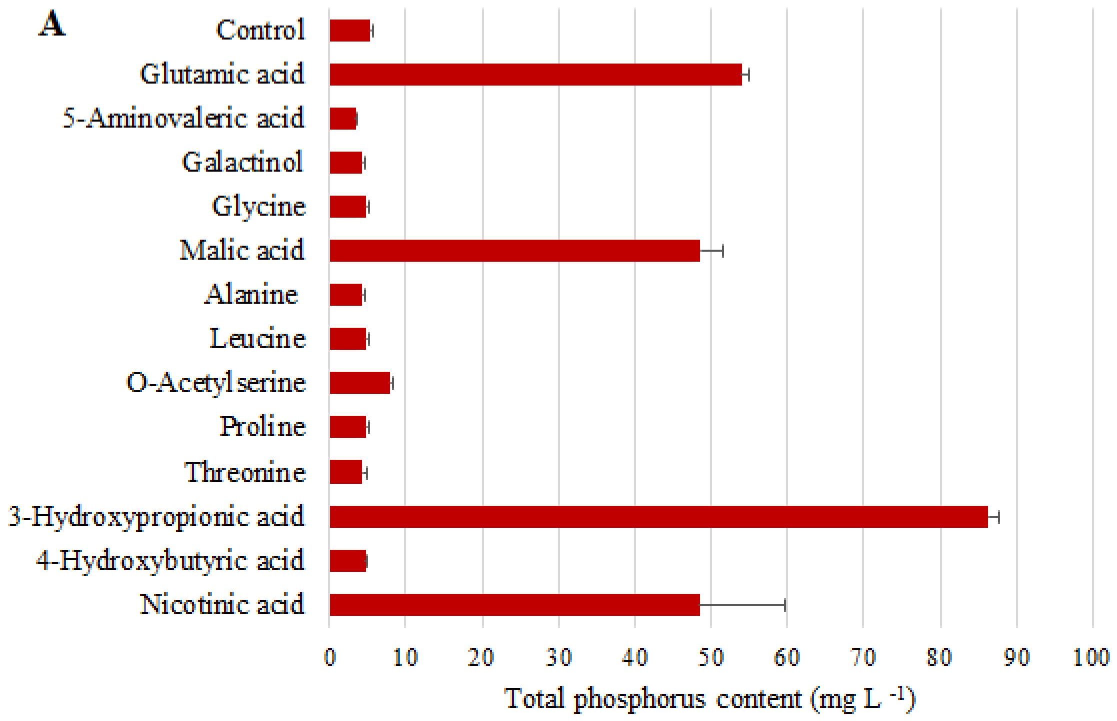
A
Control
Glutamic acid
5-Aminovaleric acid
Galactinol
Glycine
Malic acid
Alanine
Leucine
O-Acetyl serine
Proline
Threonine
3-Hydroxypropionic acid
4-Hydroxybutyric acid
Nicotinic acid
0
10
20
30
40
50
60
70
80
90
100
Total phosphorus content (mg L -1)

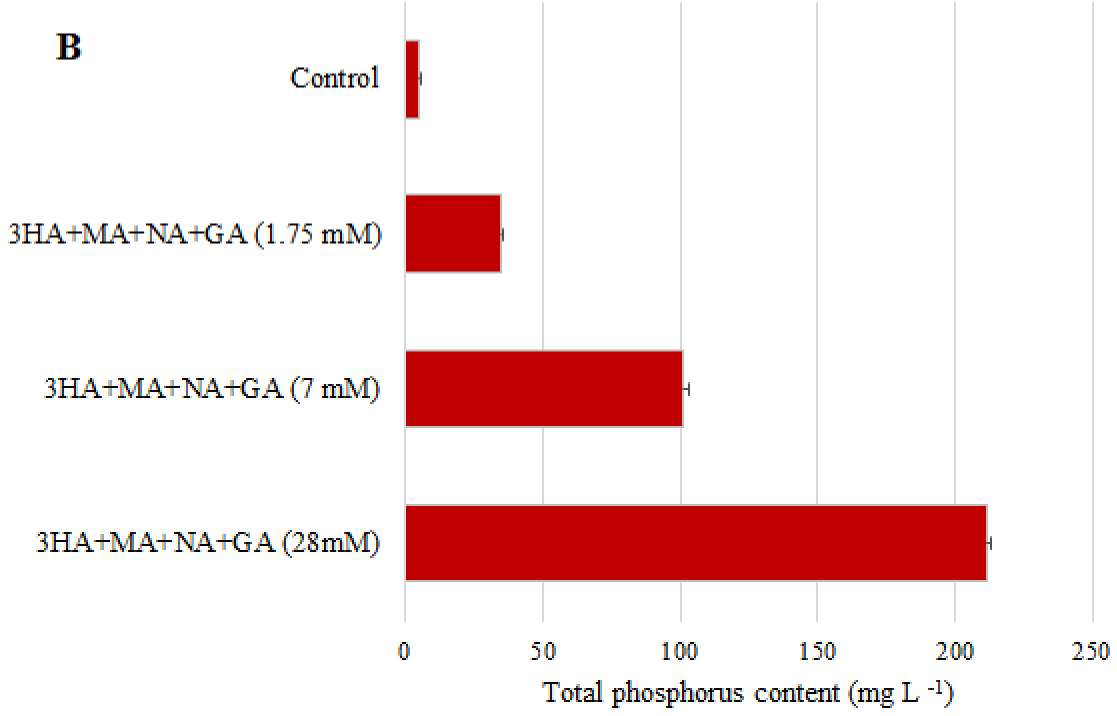
B
Control
3HA+MA+NA+GA (1.75 mM)
3HA+MA+NA+GA (7 mM)
3HA+MA+NA+GA (28mM)
0
50
100
150
200
250
Total phosphorus content (mg L -1)

**Fig 3.** Quantitative phosphorus-solubilization analyzed by Coupled Plasma-Optical Emission Spectrometer (ICP-OES) in 13 identified compounds. **(A)** Available phosphorus content in NBRIP liquid media after incubation of 72 hours with each of the 13 compounds at 7mM concentration. **(B)** Available phosphorus content in NBRIP liquid media after incubation of 72 hours from the four phosphorus-solubilizer compounds: 3-hydroxypropionic acid (3HA), malic acid (MA), nicotinic acid (NA) and glutamic acid (GA). Each compound added to the pool had 1.75 mM, 7 mM and 28 mM concentration.

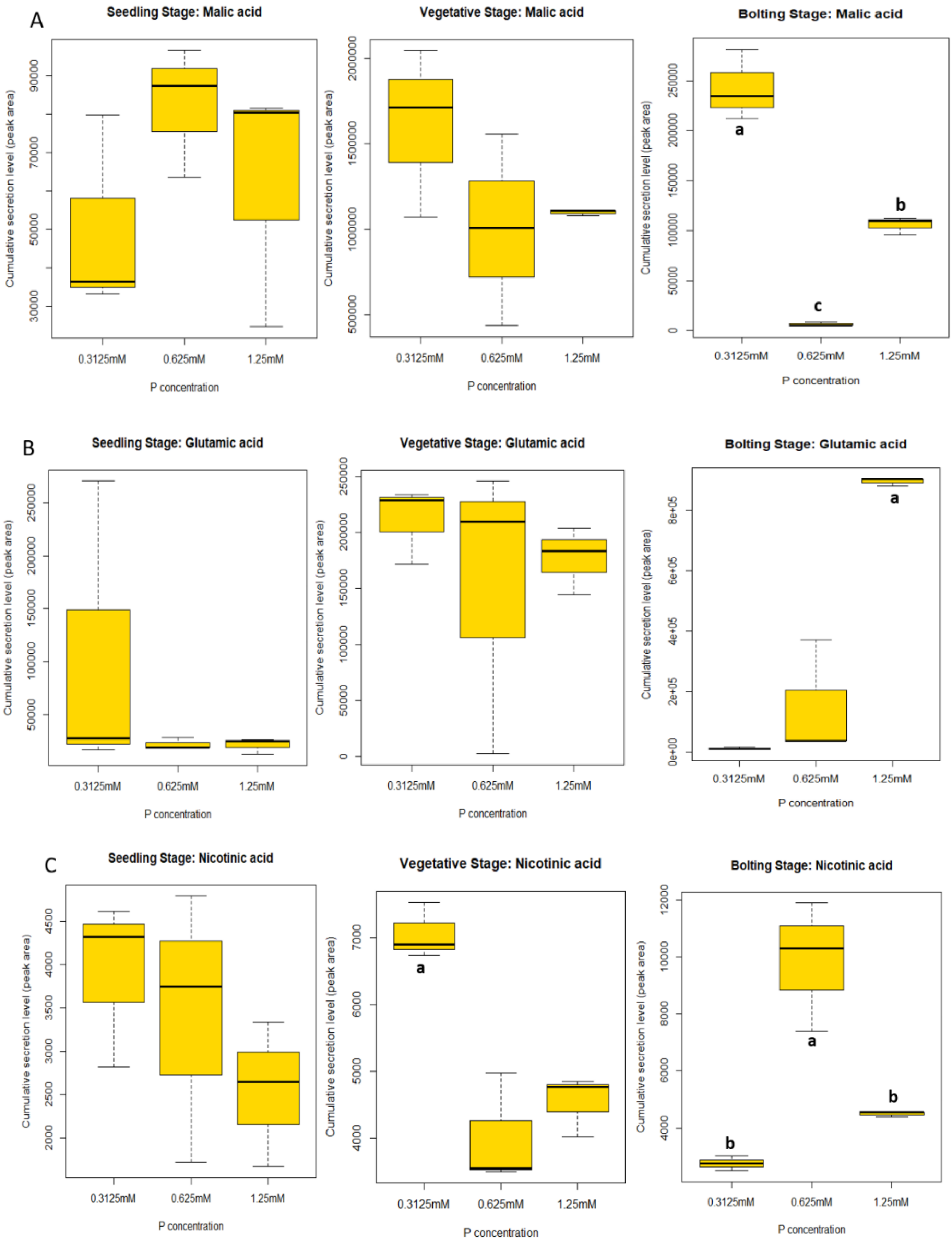

A
Seedling Stage: Malic acid
Vegetative Stage: Malic acid
Bolting Stage: Malic acid
a
b
c
B
Seedling Stage: Glutamic acid
Vegetative Stage: Glutamic acid
Bolting Stage: Glutamic acid
a
C
Seedling Stage: Nicotinic acid
Vegetative Stage: Nicotinic acid
Bolting Stage: Nicotinic acid
a
a
b
b
Cumulative secretion level (peak area)
P concentration
0.3125mM
0.625mM
1.25mM

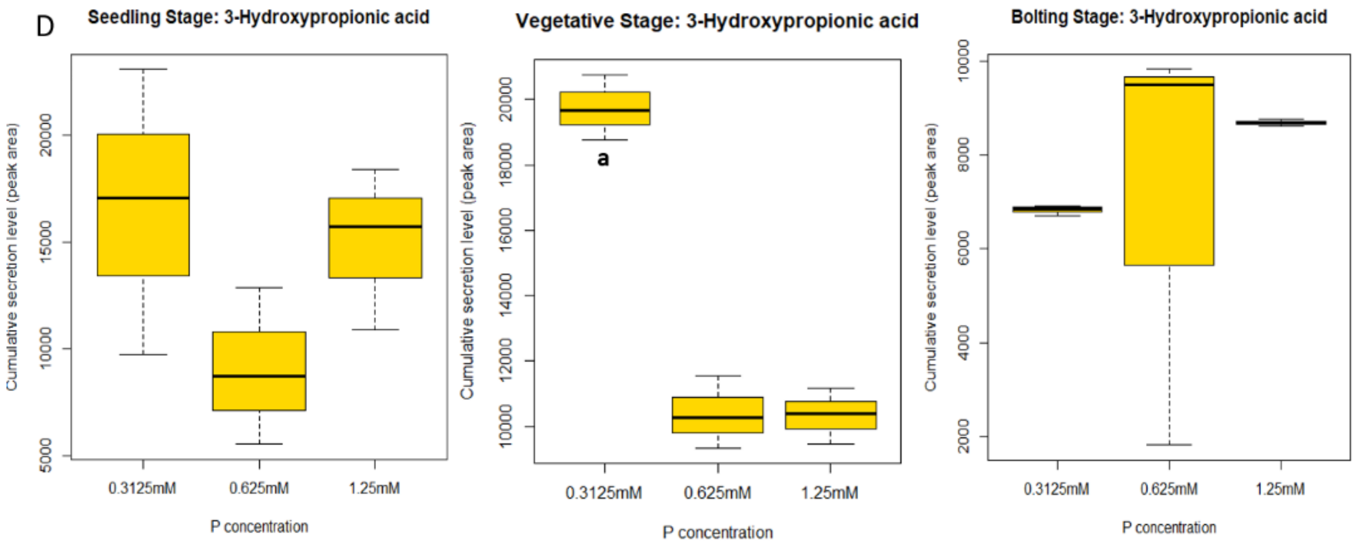


**Fig 4.** Phosphorus-solubilizer compounds showing changes in cumulative root secretion levels at three distinct developmental stages ($p<0.05$) in response to increasing phosphorus addition (0.312, 0.625 and 1.25 mM). Malic acid **(A)**, Glutamic acid **(B)**, Nicotinic acid **(C)**, 3-hydroxypropionic acid **(D)**.

Click here to access/download
**Supporting Information**
SupplementaryMaterial-PO.docx

# Role of root exudates on assimilation of phosphorus in young and old *Arabidopsis thaliana* plants

Formatted: Highlight

Hugo A. Pantigoso [1], Jun Yuan[2], Yanhui He[1,3], Qinggang Guo[1,4], Charlie Vollmer[5], Jorge M. Vivanco[1]*

[1] *Center for Rhizosphere Biology and Department of Horticulture and Landscape Architecture, Colorado State University, Fort Collins, USA*;

[2] *Jiangsu Provincial Key Lab of Organic Solid Waste Utilization, Jiangsu Collaborative Innovation Center for Organic Solid Waste Utilization, Nanjing Agricultural University, Nanjing, China*;

[3]*Key laboratory for Green Processing of Chemical Engineering of Xinjiang Bingtuan, School of Chemistry and Chemical Engineering, Shihezi University, Shihezi, China*;

[4]*Institute of Plant Protection, Hebei Academy of Agricultural and Forestry Science, Baoding, China*;

[5]*Department of Statistics, Colorado State University, Fort Collins, USA*

*Corresponding author:

E-mail: J.Vivanco@colostate.edu

# Abstract

The role of root exudates has long been recognized for its potential to improve nutrient use efficiency in cropping systems. However, studies addressing the variability of roots exudates involved in phosphorus solubilization across plant developmental stages remain scarce. In this study, we tested the effect of three increasing phosphorus fertilization levels on roots' exudate composition of *Arabidopsis thaliana* at three developmental stages (seedling, vegetative and bolting) under sterile nutrient solution-based conditions. The data ~~from root exudates~~ showed that the total exudates changed in response to the incremental addition of phosphorus starting at the vegetative level. It was found that specific metabolites decreased in quantity to phosphorus supplementation at specific stages of development. Some of those metabolites were tested for their phosphorus solubilization activity, and it was found that 3-hydroxypropionic acid, malic acid, and nicotinic acid were able to solubilize phosphorus under solid and liquid NBRIP media conditions. In summary, our data suggest that plants can release distinct compounds to deal with phosphorus deficiency needs influenced by the phosphorus nutritional status at varying developmental stages.

# Introduction

Phosphorus is an essential element for plant growth and development [1], and a non-renewable resource [2,3]. Despite the fact that the total amount of phosphorus is high in most agricultural soils, crop yields are often limited by low availability due to the non-soluble form and low mobility of this nutrient [4,5]. It has been estimated that residual phosphorus fertilizer known as 'phosphorus legacy' in soil can be sufficient to sustain crop yield for the next century and could alleviate ~~alleviating~~ expected phosphorus shortages in the next 50 years [6]. Hence, studies addressing potential solutions to exploit soil phosphorus reserves are needed.

Plants have developed several strategies for acquisition of phosphorus in low nutrient environments mainly by modifying root structure and changing the soil chemical properties in the rhizosphere [7]. These mechanisms include longer root formation and increases of root:shoot ratio allowing the transport of phosphorus from the roots to the shoots [8,9]. Certain plants such as *Lupinus* sp. can promote the formation of cluster roots to secrete phosphorus solubilizers such as citrate and malate in sufficient quantities to lower the rhizosphere pH, thus enhancing the movement of phosphorus and consequently plant uptake [10,11]. The secretion of phosphorus solubilizers is not restricted to cluster root-forming plants. Several other species such as alfalfa, spinach and radish have also been documented to increase the efflux of organic anions as a result of a lack of phosphorus available in the soil [12-14].

Increasing phosphorus solubility can also be achieved by modifying the rhizosphere chemistry. Root-secreted phosphorus solubilizers are capable of increasing solubility of a variety of insoluble phosphorus forms in the soil such as organic phosphorus, inorganic phosphorus ~~such as~~like calcium phosphate, and humic substances bonded to phosphorus anions such as Al (III) and Fe (III). They can be classified as protons or $OH^-/HCO_3^-$ equivalents, redox equivalents and

di- and tricarboxylic acid anions [13]. The mechanisms used by plants to solubilize phosphorous vary according to the plant species, nutritional status of the plant and soil and environmental conditions [15]. However, organic acids such as oxalate, citrate and malate are recurrent in a variety of plant species and thereby are the most studied means used by plants to solubilize phosphorous [13]. Considerably less research has been performed to explore the total root exudate profile and to identify other compounds exerting similar and complementary functions in the rhizosphere of *A. thaliana* across its phenological stages.

All plants share a similar need for phosphorus, but this need differs broadly based on the crop type and its developmental stage [16,17]. In general, most crops require early phosphorus supplementation for optimum yield [18]. Nevertheless, higher amounts of phosphorus in later growth stages are required proportional to increases in the biomass of the plant [19-22]. Due to the variation of phosphorus demand during the plant's lifetime, it becomes necessary to fully understand fluctuations of root exudates as a means to solubilize the phosphorus present in the P legacy of the soil.

In this study, we tested the effect of three phosphorus fertilization levels on root exudates' composition of *A. thaliana* at distinct developmental stages. We hypothesized that phosphorus amendment would negate the intrinsic ability of certain root exudate compounds to solubilize inorganic phosphorus. The results showed that the total exudates changed in response to the addition of phosphorus, and that certain metabolites were reduced under increasing phosphorus amendments at varying growth points. As a proof of concept, some of the metabolites that decreased in quantity to the phosphorus addition were tested and four of them were found to solubilize phosphorus.

# Materials and methods

## Plant growth conditions, phosphorus fertilization and collection of root exudates

*Arabidopsis thaliana* (L.) Heynh wild-type Columbia seeds were obtained from Lehle Seeds (Round Rock, USA) and surface sterilized with Clorox bleach (Sodium Hypochlorite, 8.25%) for 1 min. Seeds were rinsed with distilled water 3 times and plated on different phosphorus levels of Murashige and Skoog (MS) agar (1.5%) (supplemented with 3% sucrose) in square plates.  The plates were placed vertically in a growth chamber (Percival Scientific) at 25 ± 2 °C with a photoperiod of 16 h : 8 h, light : dark for germination. The germinated seedlings were grown in three phosphorus levels: full strength (100%, 1.25mM), half (50%, 0.625mM) and a quarter (25%, 0.3125mM) in solid MS medium as described above. The three phosphorus levels used in this study did not stimulate the plant starvation response, which is generally activated at values below 2 µM of phosphorus in soil solution [23]. Instead, we followed a low and high fertilization regime used commercially in agriculture [24]. phosphorus concentration was adjusted with $Na_2HPO_4$ and $NaH_2PO_4$, and phosphorus -free MS medium was used as basal medium. After seven days of growth, the seedlings were transferred to six well plates, each well containing 5 ml of liquid MS with 1% sucrose with one individual seedling per well containing distinct phosphorus levels as described above.  The seedlings grown in solid MS at the particular phosphorus level were placed under the same phosphorus level under liquid M conditions. All the plates were placed on an orbital shaker at 90 rpm under 25 ± 2 °C and lighted by cool white fluorescent light (45µmol $m^{-2}$ $s^{-1}$) with a photoperiod of 16 h : 8 h, light : dark. The nutrient solutions were replaced every week by transferring the plants to new six well plates with 5 ml of fresh liquid MS medium under the same phosphorus levels as stated above. Root exudates of different developmental stages were collected as follows: 1-3 days after transplanting for

seedling stage, 8-10 days after transplanting for vegetative stage, and 15-17 days after transplanting for bolting stage. Prior to the collection of root exudates, plants were removed and washed mildly with sterile water and subsequently placed in new wells containing 5 ml of sterile water for two days. The solution in which the plants were floating was collected and considered as the root exudates. The solution of one plate containing six wells with six individual plants was pooled and considered as one replicate. We used three replicates for each stage under three phosphorus levels. The root exudates were filtered through a 0.45 µm (Millipore, MA) to remove root sheathing and root border-like cells and were stored at -20 °C for Gas Chromatography-Mass Spectrometry (GC-MS) analysis.

## Gas Chromatography-Mass Spectrometry (GC-MS) of root exudates

To characterize the chemical composition, root exudates that were freeze-dried were then processed as described by Chaparro et al, [25] and subjected to Gas Chromatography-Mass Spectrometry (GC-MS) analyses at the Genome Center Core Services, University of California Davis. Extracts were briefly dried under nitrogen gas and then methoximated and trimethylsilylated. The derivatives were analyzed by an Agilent 6890 gas chromatograph (Santa Clara, CA) containing a 30-m-long, 0.25-mm inner diameter rtx5Sil-MS column with an additional 10 m integrated guard column. Metabolites were detected using the BinBase algorithm [26] and identified by comparing the retention index and mass spectrum of each analyte against the Fiehn mass spectral library from the West Coast Metabolomics Center, University of California Davis.

## Statistical analysis of total root exudate data

To discover the differential expression levels of compounds across the plant's growth stages and fertilizer levels, R statistical software (R Core Team, 2017) was used to perform Principal Components Analysis (PCA) on all annotated compounds. For each of the following analyses, we performed a PCA by first separating the total root exudate data by plant growth stage. Within each plant growth stage (e.g. Bolting), we performed a centered and scaled PCA. By verifying that the first two principal components explained a sufficient amount of the variance in the compound(s) expression levels, we were able to determine which compounds had the highest correlation with these components through the magnitude of the variance. The variances are representative of a compound at each growth stage and at a particular phosphorus level. The largest variance represents the highest correlation to the principal components. This method allowed us to determine which compounds explained most of the variance across the fertilizer levels and for each of the plant's growth stages.

Significant differences between phosphorus amendment and compound counts level per developmental stage were analyzed using a one-way ANOVA. Tukey HSD test was used to identify significance ($p < 0.05$) among phosphorus treatments.

## Qualitative analysis of phosphorus solubilizing ability of compounds derived from root exudates

From a list of selected compounds only 13 of them diluted in dd$H_2O$ at the desired concentration (100 mM) (Table 1). We used the reported concentration of organic acids in the rhizosphere (1 µM to 100 mM) as reference to select the concentration of the compounds tested in this study [27-29]. These 13 compounds were qualitatively evaluated for their phosphorus

solubilizing abilities on NBRIP (National Botanical Research Institute phosphate growth medium) solid medium containing glucose 10.0 g, $Ca_3(PO_4)_2$ 5.0 g, NaCl 0.2 g, $MgSO_4 \cdot 7H_2O$ 0.5 g, $(NH_4)_2SO_4$ 0.5 g, KCl 0.2 g, $MnSO_4$ 0.03 g, $FeSO_4 \cdot 7H_2O$ 0.003 g, agar 12 g in 1 L water, pH：7.0-8.0 [30]. All compounds used in this experiment were purchased from Thermo-Fisher Scientific. The specific solution (100 µL) of each compound was placed on NBRIP solid medium. The plates were inoculated at room temperature and let to sit overnight. phosphorus solubilizing ability was visually judged as a clear halo around every drop of solution containing the given compound.

**Table 1**. Five top compounds from root exudates identified by its spatial separation on the Principal Component Analysis (PCA). Compounds are divided by phosphorus level and plant developmental stages. (*) are compounds diluted in ddH2O at 100 mM.

| **Growth Stage** | **P level (%)** | | **Compound name** | |
|---|---|---|---|---|
| Seedling | N/A | | N/A | |
| Vegetative | 25 | 1 | Nicotinic acid | * |
| | | 2 | 4-hydroxybutyric acid | * |
| | | 3 | 3-hydroxypropionic acid | * |
| | | 4 | 1-monostearin | |
| | | 5 | 1-monopalmitin | |
| Vegetative | 50 and 100 | 6 | threonine | * |
| | | 7 | proline | * |
| | | 8 | O-acetylserine | * |
| | | 9 | Leucine | * |
| | | 10 | Alanine | * |
| Bolting | 25 | 11 | Threonic acid | |
| | | 12 | Octadecanol | |
| | | 13 | Malic acid | * |
| | | 14 | Glycine | * |
| | | 15 | Galactinol | * |
| Bolting | 50 | 16 | scopeletin | |
| | | 17 | phenylacetamine | |
| | | 18 | 5-aminovaleric acid | * |
| | | 19 | 1-monopalmitin | |
| | | 20 | 1-monoheptadecanoyl glyceride NISTT | |
| Bolting | 100 | 21 | Sophorose | |
| | | 22 | Guanine | |
| | | 23 | Glutamic acid | * |
| | | 24 | Adenine | |
| | | 25 | 1-Kestose | |

## Quantitative analysis of phosphorus solubilizing ability of water-soluble compounds (individually and combined)

For quantification analysis, 35 μL of the same concentration previously tested (100 mM) of the 13 compounds were added to 5 mL liquid NBRIP medium resulting in a final concentration of 7mM. The tubes were then placed at a continuous agitation at 150 rpm on a rotary shaker for 72 h. Afterwards, the solution was centrifuged at 6000 rpm for 5 min, and the supernatant was filtered with 0.2 µm filter (Thermo-Fisher Scientific). Liquid NBRIP medium without compound addition was used as control. The concentration of soluble phosphorus in the supernatant was analyzed accordingly with the protocol by Soltanpour et al, [31] and measured by Inductive Coupled Plasma-Optical Emission Spectrometer (ICP-OES) (Perkin Elmer 7300DV) at the Soil, Water and Plant Testing Laboratory of Colorado State University. This experiment and analysis were repeated twice with 3 repetitions.

In order to determine the potential cumulative effect of 3-hydroxypropionic acid, malic acid, nicotinic acid, and glutamic acid they were combined and the available phosphorus (mg $l^{-1}$) was determined by OES-ICP. A phosphorus gradient that included the previous tested concentration (7 mM per compound), a higher (28 mM per compound) and a lower (1.75 mM per compound) concentration was tested in order to compare if the combination of compounds would equal or surpass the effect of a single compound. Each compound added to the pool had 1.75 mM, 7 mM and 28 mM concentration. Thus, the combination effect of four compounds were tested in a liquid NBRIP medium.

## Statistical analysis for quantitative phosphate release

Significant differences between phosphorus content measured in NBRIP liquid media was analyzed using a one-way ANOVA. Tukey HSD test was used to identify significant treatments in multiple comparisons. P-value were considered significant below 0.05. Assumptions of normality and homogeneity of the data were confirmed prior to the analysis.

# Results

## Effect of phosphorus levels on *A. thaliana* root exudates at different plant developmental stages

The effect of increasing phosphorus at three different concentrations on the root exudates of *A. thaliana* was analyzed at various developmental stages. In total, 456 compounds were detected by GC-MS among the treatments. The data set was reduced to 201 annotated compounds, and only those with a proper identification were kept for statistical analysis. The grouping of the compounds in the plot maintained the same pattern even after subtracting the non-annotated compounds (S1 Fig), suggesting that all of the differentially expressed compounds were indeed annotated by the GC-MS analyses.

The variability in our data, after subtracting the non-annotated compounds, was analyzed using a principal component analysis (PCA) where variability of component 1 (PC1) accounted for 29.8%, while component 2 (PC2) accounted for 21.7%. We determined that the plant's developmental stage was responsible for the separation of the compounds in three marked groups: seedling, vegetative and bolting (Fig 1A) as previously reported [25,32,33]. When fertilizer levels (25% phosphorus, 50% phosphorus and 100 % phosphorus) were applied to each developmental stage, segregation was observed in certain developmental stages and P levels (S2 Fig). Overall, phosphorus levels did not cause a separation effect on the root exudates patterns of

seedling stage, but the event was observed in the vegetative and particularly in the bolting phases (Fig 1A). In the vegetative stage, a clear parting was observed at 25% phosphorus compared to the 50 and 100% treatments (Fig 1B). In the bolting stage, there was a clear division between the three fertilizer levels, but the 50% level was the most secluded rate (Fig 1C).

## Differences in compound-levels in the vegetative and bolting stages due to phosphorus fertilization

A separate analysis was performed to determine correlations between different and highly expressed compounds for a specific fertilization level and developmental stage. We focused our analysis on treatments 25% phosphorus at the vegetative stage, and 25%, 50% and 100% phosphorus at the bolting stage because these treatments showed to have the highest dissimilarity in the PCA. In addition, treatments 50% and 100% at the vegetative stage were grouped because they were clustered in the PCA. For each of these treatments, we found the five top compounds that explained the largest proportion of the variance in the principal components (Table 1). In addition, the expression levels of the compounds based on phosphorus level and growth stage were determined (S3-S4 Figs). We found some interesting patterns, such as that some compounds decreased expression upon increased phosphorus fertilization (e.g. 3-hydroxypropionic acid, malic acid, galactinol), while other compounds showed a positive correlation with phosphorus amendment (e.g. guanine, glutamic acid, sophorose).

## Root-exudate metabolites solubilize calcium phosphate in solid and liquid media

We then tested the ability of some of the selected compounds irrespective of their expression level upon phosphorus fertilization to induce phosphorus solubilization. We found

that 3-hydroxypropionic acid, malic acid, and nicotinic acid formed a clear halo when tested at a concentration of 100 mM, indicating the ability of this compound to solubilize phosphorus from calcium phosphate (Fig 2). All the remaining tested compounds did not present phosphorus solubilizing ability at 100 mM.

The phosphorus -solubilizing effect of the selected compounds was further tested using a more sensitive technique (OES-ICP) at a final concentration of 7 mM in liquid NBRIP medium. The results showed that in addition to 3-hydroxypropionic acid, malic acid, and nicotinic acid, glutamic acid was also found to express phosphorus solubilizing ability on sterile nutrient solution using spectroscopy measurements. The results showed that the soluble P in the medium without compound addition was 5.34 mg $l^{-1}$ (control) compared to 86.38, 48.61, 48.46, and 53.92 mg $l^{-1}$ when 3-hydroxypropionic acid, malic acid, nicotinic acid, and glutamic acid were added (Fig 3A).

Further analysis aimed to test the combined effect of all the four compounds on NBRIP medium resulting in 34.5 (for 1.75 mM) 101.21 (for 7mM) and 211.63 (for 28 mM) mg $l^{-1}$ of available P compared to 4.69 mg $l^{-1}$ from the control (Fig 3B).

## The plant mediates changes in levels of phosphorus solubilizer compounds in response to phosphorus status and developmental stage

We further analyzed the expression levels upon phosphorus fertilization of 3-hydroxypropionic acid, malic acid, nicotinic acid, and glutamic acid in the different developmental stages. Depending on the compound, our results showed that the cumulative secretion levels of the compounds increased, decreased or remained statistically similar ($p>0.05$)

following increasing levels of phosphorus amendment (Fig 4). At the seedling stage, changes in cumulative secretion for the four phosphorus solubilizers were not statistically significant, which agrees with the PCA analysis (Fig 1A). Interestingly, at the vegetative stage nicotinic acid and 3-hydroxypropionic acid showed higher abundance at the lowest phosphorus level (0.3125 mM) and decreased significantly ($p<0.05$) for the two higher P levels (0.625 and 1.25 mM). Malic and glutamic acid followed a similar pattern; however, their changes were not statistically significant. At the bolting stage, differences of compound cumulative secretion were observed for glutamic acid, malic acid and nicotinic acid, but not for 3-hydroxypropionic acid which did not increase or decrease following an incremental phosphorus level. Cumulative secretion of malic acid was reduced significantly from 0.3125 to 0.625 mM phosphorus treatments, and then incremented its cumulative secretion for the highest phosphorus treatment (1.25 mM); however, cumulative secretion of malic acid for the two highest phosphorus levels (0.625 and 1.25 mM) were below the values at the lowest phosphorus rate (0.3125 mM) (Fig 4A). Similarly, cumulative secretion of nicotinic acid increased significantly from 0.3125 to 0.625 mM but dropped for 1.25 mM of phosphorus (Fig 4C). Lastly, glutamic acid consistently increased upon higher phosphorus fertilization reaching its peak at 1.25 mM of phosphorus (Fig 4B).

# Discussion

For the most part, the influence of phosphorus fertilization on root secretion has been studied at specific developmental stages [34,35], among genotypes of the same plant species [36] or between different species [37]. For instance, Vengavasi et al. [36] found cultivar-dependent differences in root exudation of soybeans grown under phosphorus -sufficient versus phosphorus -deficient conditions. It is worth noting that these plants were sampled at the reproductive stage

of growth, a metabolically active stage with higher demand for energy and phosphorus nutrition. In a different study, the authors found differences in root exudation in maize seedlings grown in phosphorus -sufficient and phosphorus -deficient conditions [34]. In contrast, here we studied the interplay of increasing phosphorus fertilization on root exudation at different plant developmental stages (seedling, vegetative and bolting) in *A. thaliana*.

Our results showed that the root exudate profiles were similar within the seedling stage across all phosphorus fertilization treatments (Fig 1A). Thus, we hypothesized that at this growth stage roots did not respond to phosphorus fertilization. *A. thaliana* is considered a plant that can thrive in marginal soils where optimum nutrient conditions are limited [38], and at early developmental stages the plant does not require high amounts of phosphorus as a mechanism to cope with poor soil conditions [39]. In fact, it has been shown that the reserves of phosphorus in the seeds of several plants, including *Brassicaceae* species, can support seedling growth in a medium lacking phosphorus for at least four weeks after germination [40-42,39]. In contrast, maize, a monocotyledonous plant, possesses two genes induced by phosphate starvation in its genomes compared to five in dicotyledonous such as *A. thaliana* [43,44]; suggesting a difference in phosphorus responsiveness between these two plant groups. In addition, signs of phosphorus scarcity in dicotyledonous (e.g. *A. thaliana*) is often observed at later stages of ~~phosphorus deficiency~~ growth [45]. In the vegetative stage, the root exudates at 50% and 100% phosphorus showed greater visual similarity in the PCA than the exudates at 25% phosphorus, evidencing an initial sensing from the plant in response to its phosphorus demand. At the bolting stage, the three amounts of phosphorus fertilization showed distinct root exudation patterns. These results may suggest that as the plant ages the demand for phosphorus increases, as evidenced by the differential root exudation profiles [46]. This result is in accordance with Tawaraya et al. [47],

who showed that phosphorus content increased in shoot and root-dried soybean tissues as the plant developed, and that root exudates' content, collected on day 1, 5, 10 and 15 of growth, increased for phosphorus-depleted compared to phosphorus -sufficient treatments.

Our results suggest that plants in the vegetative stage only sense the 25% phosphorus as low levels, whereas plants at bolting stage sense both 25% and 50% phosphorus as low. This pattern of incremental phosphorus requirement during progression in plant development is common in annual plants such as *A. thaliana* [39]. For instance, *Brassica napus* L. requires an incremental supply of phosphorus at flowering onset, which is critical for protein and oil synthesis, and the development of seeds [48,49]. It is worth noting that the intermediate phosphorus rate (50%) at bolting stage was largely secluded (in the PCA plot) compared to 25% and 100% treatments, indicating a higher dissimilarity in root exudation composition. This could be due to the fact that the functions of plant metabolites are diverse and are not restricted only to nutrient acquisition. For instance, root exudates can be substrates, chemotactic or signaling molecules that regulate plant root and microbial interactions [50]. Such plant modulation can be specific to developmental stages [51].

The PCA data allowed for the visual determination of changes in root exudation composition between plant developmental stages and phosphorus fertilization levels. We then developed a list of compounds based on those differences observed in the PCA in response to phosphorus nutrient status; and four of those compounds (i.e. 3-hydroxypropionic acid, nicotinic acid, glutamic acid and malic acid) were confirmed as phosphorus solubilizers. Three (3-hydroxypropionic acid, nicotinic acid, and malic acid) out of the four compounds were significantly more abundant at the lowest phosphorus rate and reduced in concentration as phosphorus amendments were elevated. Our findings agree with a variety of studies evaluating

the exudation of organic acids (e.g. malic acid) in different plant species under various phosphate availabilities [52,13]. Malic acid is a primary compound released by roots under phosphorus deficiency, but often not the most effective [52]. ~~While~~ In contrast, nicotinic acid and glutamic acid are less abundant than malic acid or oxalic acid in plant species ~~but reported in crops such as rice and soybeans~~ [46,53]. 3-~~H~~hydroxypropionic acid has not been previously associated with phosphorus solubilization, however it has been described as a natural product of a plant endophytic fungus [54,55]. Further, the understanding of the dynamic of these compounds throughout plant phenology is scarce mainly because these studies are often performed in hydroponic systems limiting the root exudate collection to early stages of plant development for a variety of plant crops [52,56]. In our study, we observed that the cumulative secretion of malic acid significantly increased during low phosphorus availability, but it was restricted to the bolting stage ~~only~~. 3-~~H~~hydroypropionic acid and nicotinic acid followed the same pattern, however they were significantly expressed only the in vegetative stage. Conversely, glutamic acid and nicotinic acid increased in abundance at bolting stage when phosphorus levels increased. Interestingly, nicotinic acid switched cumulative secretion depending on the developmental stage. It has been reported that nicotinic acid induced flowering in *Lemna* ~~L.~~ plants [57], and that nicotinic acid can alleviate abiotic plant stresses by increasing phytohormones levels such as indol-3-acetic acid (IAA) and gibberellic acid ($GA_3$) [58].

Based on these observations we hypothesize that plant developmental stage modulates root exudation to deal with phosphorus deficiency by three potential mechanisms: (1) Plants secrete synergistic phosphorus-solubilizing compounds in stages of high P demand. In this study nicotinic acid, a moderate phosphorus -solubilizer, was released in combination with 3-hydroxypropionic acid, a stronger phosphorus-solubilizer, improving phosphorus acquisition at

the vegetative stage (see Fig 3). This agrees with a recent study showing synergistic association of citrate and phytase to improve acquisition of plant unavailable phosphorus in tobacco in the vegetative stage [59]. (2) Plants prevent the degradation of phosphorus-solubilizer compounds such as malic acid, rapidly degraded by soil microbes [60], by releasing a different compound such as 3-hydroxypropionic acid preceding a growth stage of high phosphorus demand. It has been shown that certain plants, such as lupin, can release compounds that inhibit microbial activity to reduce organic acid degradation prior to the release of organic acids. [61]. Lastly, (3) p~~P~~lants secrete~~s~~ specific compounds to mediate either direct nutrient solubilization or the proliferation of distinct microbial taxa (with phosphorus solubilizing activity) at specific growth stages (e.g. vegetative, bolting). In support of this hypothesis, root exudates can promote the activity of symbiotic microbes, such as phosphorus solubilizing bacteria and siderophore-releasing bacteria and exert mobilization of non-available plant nutrients in soils at a single growth stage [62-64].

It has been estimated that organic acids constitute 5 to 10 % of the total organic carbon in soil solution. The concentration of organic anions measured in the soil solution usually range from 100 nM to more than 580 uM in the rhizosphere of cluster roots [65]. However, millimolar concentrations of organic anions are likely required in the soil solution to effectively increase soluble P concentration especially in calcareous soils [66, 67]. Strom et al. [66] tested three organic acids (citrate, malic and oxalate) and a wide range of concentrations (1 mM to 100 mM) to evaluate its effects on the mobilization of phosphorus in calcareous soil. The results showed that the phosphorus mobilization of the tested compounds has a low efficiency and its effect varies depending on the type of organic acid, compound concentration, and pH. Further, due to the low phosphorus mobilization efficiency of those compounds is still argued if the benefit of

releasing large amounts of organic acids will exceed the cost of carbon lost by the plant, which can be seen as an unnecessary trade-off [66]. However, low efficiency organic acids can be particularly important in phosphorus mobilization for calcareous soils with a limited phosphorus availability for plants. Finally, our evidence supports the above-mentioned hypothesis, that plants release a combination of compounds with different phosphorus-solubilizing efficiencies, at specific stages of growth, to deal with particular phosphorus needs.

Lastly, root exudates from liquid culture systems allow the determination of exudation rates unaltered by soil matrix or microbial decomposition if performed under sterile conditions as we did in this study [56]. However, the quality and quantity of the root exudate profile may be impacted by the nutrient solution culture method (also known as hydroponic methods) [68]. Soil-hydroponic hybrids methods for root exudation collection are not exempt of potential physical/physiological perturbances. Thus, nutrient solution culture methods remain especially important to assess temporal dynamics of root exudates.

In summary, the significance of these findings relies on the potential of customizing specific metabolites to be utilized as soil amendments under the most limiting phosphorus conditions and most demanding stage of plant growth. The role of secondary metabolites in phytoremediation efforts has been previously investigated [69] as well as the use of customized synthetic bacteria communities to modify plant phosphate accumulation [70,71]. However, the use of customized metabolites for phosphorus acquisition remains unexplored.

# Conclusions

The data collected indicate that root exudate patterns change as a response to the supply levels of phosphorus, and this change was accentuated as the plant reached maturity, when

phosphorus demands are higher. 3-H~~h~~ydroxypropionic acid (3-HA) and nicotinic acid accumulated significantly at the vegetative stage under lower phosphorus supplementation and was found to solubilize phosphorus under both solid and liquid medium. This study sheds light on the influence of plant nutrient status and plant phenological growth stages driving the composition of plant root exudates. Future research should focus on understanding the effects of metabolites at a particular developmental stage of growth under phosphorus depleted soil conditions, as well as to test the potential of these phosphorus solubilizer compounds in making phosphorus available for plants ~~under~~ grown in soils saturated in unavailable phosphorus forms ~~in agricultural lands~~.

# Conflict of interest

The authors declare no competing interests.

# Acknowledgment

We thank Dr. James Self, manager of the Soil, Water and Plant testing laboratory at Colorado State University for their invaluable advice regarding phosphorous chemical analysis. J. Y. was supported by National Postdoctoral Program for Innovative Talents (BX201600075) and Natural Science Foundation of Jiangsu Province (BK20170724), Y. H. was supported by China Scholarship Council (No. 201709505007).

# References


1. Lynch JP. Roots of the second green revolution. Australian Journal of Botany. 2007 Sep 7;55(5):493-512.
2. Cordell D, Drangert JO, White S. The story of phosphorus: global food security and food for thought. Global environmental change. 2009 May 1;19(2):292-305.
3. Gilbert N. The disappearing nutrient: phosphate-based fertilizers have helped spur agricultural gains in the past century, but the world may soon run out of them. Natasha Gilbert investigates the potential phosphate crisis. Nature. 2009 Oct 8;461(7265):716-9.
4. Torri SI, Correa RS, Renella G. Biosolid application to agricultural land—A contribution to global phosphorus recycle: A review. Pedosphere. 2017 Feb 1;27(1):1-6.
5. Wei Y, Zhao Y, Wang H, Lu Q, Cao Z, Cui H, Zhu L, Wei Z. An optimized regulating method for composting phosphorus fractions transformation based on biochar addition and phosphate-solubilizing bacteria inoculation. Bioresource technology. 2016 Dec 1;221:139-46.
6. Zhu J, Li M, Whelan M. Phosphorus activators contribute to legacy phosphorus availability in agricultural soils: A review. Science of the Total Environment. 2018 Jan 15;612:522-37.
7. Neumann G, Romheld V. The release of root exudates as affected by the plant's physiological status. InThe rhizosphere 2000 Nov 17 (pp. 57-110). CRC press.
8. Bates TR, Lynch JP. Root hairs confer a competitive advantage under low phosphorus availability. Plant and Soil. 2001 Oct 1;236(2):243-50.
9. Heydari MM, Brook RM, Jones DL. The role of phosphorus sources on root diameter, root length and root dry matter of barley (Hordeum vulgare L.). Journal of plant nutrition. 2019 Jan 2;42(1):1-5.

10. Braum SM, Helmke PA. White lupin utilizes soil phosphorus that is unavailable to soybean. Plant and soil. 1995 Sep 1;176(1):95-100.
11. Hocking PJ, Jeffery S. Cluster-root production and organic anion exudation in a group of old-world lupins and a new-world lupin. Plant and Soil. 2004 Jan 1;258(1):135-50.
12. Lipton DS, Blanchar RW, Blevins DG. Citrate, malate, and succinate concentration in exudates from P-sufficient and P-stressed Medicago sativa L. seedlings. Plant Physiology. 1987 Oct 1;85(2):315-7.
13. Gerke J. The acquisition of phosphate by higher plants: effect of carboxylate release by the roots. A critical review. Journal of Plant Nutrition and Soil Science. 2015 Jun;178(3):351-64.
14. Zhang FS, Ma J, Cao YP. Phosphorus deficiency enhances root exudation of low-molecular weight organic acids and utilization of sparingly soluble inorganic phosphates by radish (Raghanus satiuvs L.) and rape (Brassica napus L.) plants. Plant and Soil. 1997 Oct 1;196(2):261-4.
15. Hinsinger P. Bioavailability of soil inorganic P in the rhizosphere as affected by root-induced chemical changes: a review. Plant and soil. 2001 Dec 1;237(2):173-95.
16. Marschner H. Soil- Root Interface: Biological and Biochemical Processes. Soil chemistry and ecosystem health. 1998 Jan 1;52:191-231.
17. Marschner H., 1995. Nutrient availability in soils. In: Marschner, editors. Mineral Nutrition of Higher Plants. London, UK: Academic Press. p. 483-507.
18. Grant CA, Flaten DN, Tomasiewicz DJ, Sheppard SC. The importance of early season phosphorus nutrition. Canadian Journal of Plant Science. 2001 Apr 1;81(2):211-24.
19. Marti HR, Mills HA. Nutrient uptake and yield of sweet pepper as affected by stage of development and N form. Journal of plant nutrition. 1991 Nov 1;14(11):1165-75.

20. Marti HR, Mills HA. Calcium uptake and concentration in bell pepper plants as influenced by nitrogen form and stages of development. Journal of plant nutrition. 1991 Nov 1;14(11):1177-85.

21. Awonaike KO, Kumarasinghe KS, Danso SK. Nitrogen assimilation and distribution in field-grown cowpea at various growth stages. Soil Science Society of America Journal. 1991;55(1):81-5.

22. White PJ, Veneklaas EJ. Nature and nurture: the importance of seed phosphorus content. Plant and soil. 2012 Aug 1;357(1-2):1-8.

23. Föhse D, Claassen N, Jungk A. Phosphorus efficiency of plants. Plant and soil. 1988 Aug 1;110(1):101-9.

24. Sattari SZ, Bouwman AF, Giller KE, van Ittersum MK. Residual soil phosphorus as the missing piece in the global phosphorus crisis puzzle. Proceedings of the National Academy of Sciences. 2012 Apr 17;109(16):6348-53.

25. Chaparro JM, Badri DV, Bakker MG, Sugiyama A, Manter DK, Vivanco JM. Root exudation of phytochemicals in Arabidopsis follows specific patterns that are developmentally programmed and correlate with soil microbial functions. PloS one. 2013;8(2).

26. Fiehn O, Wohlgemuth G, Scholz M, Kind T, Lee DY, Lu Y, Moon S, Nikolau B. Quality control for plant metabolomics: reporting MSI- compliant studies. The Plant Journal. 2008 Feb;53(4):691-704.

27. Jones DL. Organic acids in the rhizosphere–a critical review. Plant and soil. 1998 Aug 1;205(1):25-44.

28. Veneklaas EJ, Stevens J, Cawthray GR, Turner S, Grigg AM, Lambers H. Chickpea and white lupin rhizosphere carboxylates vary with soil properties and enhance phosphorus uptake. Plant and Soil. 2003 Jan 1;248(1-2):187-97.

29. Ström L, Owen AG, Godbold DL, Jones DL. Organic acid behaviour in a calcareous soil implications for rhizosphere nutrient cycling. Soil Biology and Biochemistry. 2005 Nov 1;37(11):2046-54.

30. Nautiyal CS. An efficient microbiological growth medium for screening phosphate solubilizing microorganisms. FEMS microbiology Letters. 1999 Jan 1;170(1):265-70.

31. Soltanpour PN, Jones Jr JB, Workman SM. Optical emission spectrometry. Methods of Soil Analysis: Part 2 Chemical and Microbiological Properties. 1983 Feb 1;9:29-65.

32. Chaparro JM, Badri DV, Vivanco JM. Rhizosphere microbiome assemblage is affected by plant development. The ISME journal. 2014 Apr;8(4):790-803.

33. Yuan J, Chaparro JM, Manter DK, Zhang R, Vivanco JM, Shen Q. Roots from distinct plant developmental stages are capable of rapidly selecting their own microbiome without the influence of environmental and soil edaphic factors. Soil Biology and Biochemistry. 2015 Oct 1;89:206-9.

34. Carvalhais LC, Dennis PG, Fedoseyenko D, Hajirezaei MR, Borriss R, von Wirén N. Root exudation of sugars, amino acids, and organic acids by maize as affected by nitrogen, phosphorus, potassium, and iron deficiency. Journal of Plant Nutrition and Soil Science. 2011 Feb;174(1):3-11.

35. Keerthisinghe G, Hocking PJ, Ryan PR, Delhaize E. Effect of phosphorus supply on the formation and function of proteoid roots of white lupin (Lupinus albus L.). Plant, Cell & Environment. 1998 May;21(5):467-78.

36. Vengavasi K, Pandey R. Root exudation potential in contrasting soybean genotypes in response to low soil phosphorus availability is determined by photo-biochemical processes. Plant Physiology and Biochemistry. 2018 Mar 1;124:1-9.
37. Li L, Li SM, Sun JH, Zhou LL, Bao XG, Zhang HG, Zhang FS. Diversity enhances agricultural productivity via rhizosphere phosphorus facilitation on phosphorus-deficient soils. Proceedings of the National Academy of Sciences. 2007 Jul 3;104(27):11192-6.
38. Mitchell-Olds T, Schmitt J. Genetic mechanisms and evolutionary significance of natural variation in Arabidopsis. Nature. 2006 Jun;441(7096):947-52.
39. White PJ, Veneklaas EJ. Nature and nurture: the importance of seed phosphorus content. Plant and soil. 2012 Aug 1;357(1-2):1-8.
40. Nadeem M, Mollier A, Morel C, Vives A, Prud'homme L, Pellerin S. Relative contribution of seed phosphorus reserves and exogenous phosphorus uptake to maize (Zea mays L.) nutrition during early growth stages. Plant and Soil. 2011 Sep 1;346(1-2):231-44.
41. Nadeem M, Mollier A, Morel C, Vives A, Prud'homme L, Pellerin S. Maize (Zea mays L.) endogenous seed phosphorus remobilization is not influenced by exogenous phosphorus availability during germination and early growth stages. Plant and soil. 2012 Aug 1;357(1-2):13-24.
42. Bolland MD, Baker MJ. High phosphorus concentrations in seed of wheat and annual medic are related to higher rates of dry matter production of seedlings and plants. Australian Journal of Experimental Agriculture. 1988;28(6):765-70.
43. Yang H, Knapp J, Koirala P, Rajagopal D, Peer WA, Silbart LK, Murphy A, Gaxiola RA. Enhanced phosphorus nutrition in monocots and dicots over- expressing a phosphorus-responsive type I H+- pyrophosphatase. Plant biotechnology journal. 2007 Nov;5(6):735-45.

44. Hasan MM, Hasan MM, da Silva JA, Li X. Regulation of phosphorus uptake and utilization: transitioning from current knowledge to practical strategies. Cellular & molecular biology letters. 2016 Dec;21(1):7.
45. Johnson JF, Vance CP, Allan DL. Phosphorus deficiency in Lupinus albus (altered lateral root development and enhanced expression of phosphoenolpyruvate carboxylase). Plant physiology. 1996 Sep 1;112(1):31-41.
46. Neumann G, Massonneau A, Martinoia E, Römheld V. Physiological adaptations to phosphorus deficiency during proteoid root development in white lupin. Planta. 1999 May 1;208(3):373-82.
47. Tawaraya K, Horie R, Shinano T, Wagatsuma T, Saito K, Oikawa A. Metabolite profiling of soybean root exudates under phosphorus deficiency. Soil science and plant nutrition. 2014 Sep 3;60(5):679-94.
48. Lickfett T, Matthäus B, Velasco L, Möllers C. Seed yield, oil and phytate concentration in the seeds of two oilseed rape cultivars as affected by different phosphorus supply. European Journal of Agronomy. 1999 Nov 1;11(3-4):293-9.
49. Rose TJ, Rengel Z, Ma Q, Bowden JW. Post-flowering supply of P, but not K, is required for maximum canola seed yields. European journal of agronomy. 2008 Apr 1;28(3):371-9.
50. Venturi V, Keel C. Signaling in the rhizosphere. Trends in plant science. 2016 Mar 1;21(3):187-98.
51. He Y, Pantigoso HA, Wu Z, Vivanco JM. Co- inoculation of Bacillus sp. and Pseudomonas putida at different development stages acts as a biostimulant to promote growth, yield and nutrient uptake of tomato. Journal of applied microbiology. 2019 Jul;127(1):196-207.

52. Wang Y, Lambers H. Root-released organic anions in response to low phosphorus availability: recent progress, challenges and future perspectives. Plant and Soil. 2019 Feb 12:1-22.
53. Tawaraya K, Horie R, Saito A, Shinano T, Wagatsuma T, Saito K, Oikawa A. Metabolite profiling of shoot extracts, root extracts, and root exudates of rice plant under phosphorus deficiency. Journal of plant nutrition. 2013 Jan 1;36(7):1138-59.
54. Schwarz M, Köpcke B, Weber RW, Sterner O, Anke H. 3-Hydroxypropionic acid as a nematicidal principle in endophytic fungi. Phytochemistry. 2004 Aug 1;65(15):2239-45.
55. Gunatilaka AL. Natural products from plant-associated microorganisms: distribution, structural diversity, bioactivity, and implications of their occurrence. Journal of natural products. 2006 Mar 24;69(3):509-26.
56. Oburger E, Jones DL. Sampling root exudates–mission impossible?. Rhizosphere. 2018 Jun 1;6:116-33.
57. Fujioka S, Yamaguchi I, Murofushi N, Takahashi N, Kaihara S, Takimoto A, Cleland CF. The influence of nicotinic acid and plant hormones on flowering in Lemna. Plant and cell physiology. 1986 Jan 1;27(1):109-16.
58. El-Bassiouny HM. Physiological responses of wheat to salinity alleviation by nicotinamide and tryptophan. International Journal of Agriculture and Biology. 2005;7(4):653-9.
59. Giles CD, George TS, Brown LK, Mezeli MM, Richardson AE, Shand CA, Wendler R, Darch T, Menezes-Blackburn D, Cooper P, Stutter MI. Does the combination of citrate and phytase exudation in Nicotiana tabacum promote the acquisition of endogenous soil organic phosphorus?. Plant and Soil. 2017 Mar 1;412(1-2):43-59.

60. Jones DL. Organic acids in the rhizosphere–a critical review. Plant and soil. 1998 Aug 1;205(1):25-44.

61. Weisskopf L, ABOU- MANSOUR EL, Fromin N, Tomasi N, Santelia D, Edelkott I, Neumann G, Aragno M, Tabacchi R, Martinoia E. White lupin has developed a complex strategy to limit microbial degradation of secreted citrate required for phosphate acquisition. Plant, Cell & Environment. 2006 May;29(5):919-27.

62. O'Banion BS, O'Neal L, Alexandre G, Lebeis SL. Bridging the gap between single-strain and community-level plant-microbe chemical interactions. Molecular Plant-Microbe Interactions. 2020 Feb 15;33(2):124-34.

63. Rolfe SA, Griffiths J, Ton J. Crying out for help with root exudates: adaptive mechanisms by which stressed plants assemble health-promoting soil microbiomes. Current opinion in microbiology. 2019 Jun 1;49:73-82.

64. Voges MJ, Bai Y, Schulze-Lefert P, Sattely ES. Plant-derived coumarins shape the composition of an Arabidopsis synthetic root microbiome. Proceedings of the National Academy of Sciences. 2019 Jun 18;116(25):12558-65.

65. Jones DL. Organic acids in the rhizosphere–a critical review. Plant and soil. 1998 Aug 1;205(1):25-44.

66. Ström L, Owen AG, Godbold DL, Jones DL. Organic acid behaviour in a calcareous soil implications for rhizosphere nutrient cycling. Soil Biology and Biochemistry. 2005 Nov 1;37(11):2046-54.

67. Ryan PR, Delhaize E, Jones DL. Function and mechanism of organic anion exudation from plant roots. Annual review of plant biology. 2001 Jun;52(1):527-60.

68. Oburger E, Dell'mour M, Hann S, Wieshammer G, Puschenreiter M, Wenzel WW. Evaluation of a novel tool for sampling root exudates from soil-grown plants compared to conventional techniques. Environmental and experimental botany. 2013 Mar 1;87:235-47.
69. Singer AC, Crowley DE, Thompson IP. Secondary plant metabolites in phytoremediation and biotransformation. TRENDS in Biotechnology. 2003 Mar 1;21(3):123-30.
70. Paredes SH, Gao T, Law TF, Finkel OM, Mucyn T, Teixeira PJ, Gonzalez IS, Feltcher ME, Powers MJ, Shank EA, Jones CD. Design of synthetic bacterial communities for predictable plant phenotypes. PLoS biology. 2018 Feb;16(2).
71. Awasthi A. Field-Specific Microbial Consortia Are Feasible: A Response to Kaminsky et al. Trends in biotechnology. 2019 Jun 1;37(6):569-72.

**Reviewers responses:**

**Line 1. Title. Your title is difficult to parse. Please rewrite. For example: "On the role of root exudates on the assimilation of phosphorus in young and old roots".**

**RESPONSE:** Thank you. As suggested, the title was rewritten and now it reads as follow: 'Role of root exudates on assimilation of phosphorus in young and old *Arabidopsis thaliana* plants

**Line 26, and elsewhere. Please spell out phosphorus everywhere in the text. Do not use "P". I am aware that many authors use this abbreviation. However repeated use is a poor argument for validity. In fact, abbreviations and acronyms make a text difficult to read. Abbreviations rarely occur in newspapers or fiction. Any time a reader encounters a symbol (P is a symbol for phosphorus), they must translate that symbol into words. Translation takes mental energy away from comprehension. Translation slows down the reader and gives nothing in return. It is one thing to have to write about a chemical with a name that is 38 syllables long. In that case, the name is just as difficult to read as the acronym. However phosphorus is a good word of the English language. In fact, phosphorus literally means 'carrier of light'. Rather beautiful. Spell it out.**

**RESPONSE:** Thanks for the suggestion and wonderful comments particularly related to "carrier of light". The abbreviation for phosphorus was changed for the full word throughout the manuscript.

**Line 26. "… have not been conducted…" This wording implies that you have read every paper ever published. Use less extreme wording, such as "…rarely if ever…"**

**RESPONSE:** Thanks for the suggestion. We have changed 'have not been conducted' for 'remain scarce'.

**Line 29. You are using the word 'metabolome', to refer to root exudates. This is misleading and confusing. The word metabolome refers to all of the metabolites in the cell or organism. However here, you are not measuring the metabolome. Instead you are measuring exudates. These compounds number about a dozen, far less than the threshold for 'omics'. Please remove the word 'metabolome' throughout the paper and instead talk about 'total exudates' or the equivalent. For example, the sentence staring at line 29 can read: "The composition of root exudates changed in response…"**

**RESPONSE:** Thanks. We agreed with the editor. We replaced the word metabolome for total root exudates and/or total exudate profile throughout the manuscript.

**Lines 29 and 34. You write 'in vitro' conditions. What does this mean? Instead say what the conditions are. By the way, typically in vitro implies isolated components, cells or cell fragments. I have never seen in vitro used for whole organisms. Also you write 'solid' and 'liquid media conditions'. Again, be specific, say what the solid and liquid media are.**

**RESPONSE:**

Thank you for the recommendation. To make the distinction, we have replaced the word in vitro for 'sterile nutrient solution'.

The type of media described as solid and liquid is a microbiological growth medium for screening phosphate solubilizing microorganisms known as NBRIP (National Botanical Research Institute's phosphate) growth media. This information appears in the material and methods. In line 29-34 (now 36) we have specified the type of liquid and solid media and it reads now as: "solid and liquid **NBRIP** media conditions".

**Line 31. What does 'respond negatively' mean? If you mean decreased in quantity, just say so.**

**RESPONSE:** Thank you. Changes were made accordingly and now the sentence reads as follows: **'**It was found that specific metabolites **decreased in quantity** related to phosphorus supplementation at specific stages of development.'

**Line 66. Based on long-established rules for scientific nomenclature, the word "*Arabidopsis*" (Capital A, italic font) means the genus. No matter how many illiterate molecular biologists make this mistake, rules of taxonomic nomenclature remain in force until the international committee decrees otherwise. Longstanding practice allows "arabidopsis" (lower case a, Roman font) as the common name of our friendly lab weed; however, many journals will auto-correct this by adding a capital and italics. I am not sure about PLoS One. Thus, either use 'arabidopsis' and hope the journal lets it stand, or use *A. thaliana*(italics) (but *Arabidopsis thaliana*at first mention).**

**RESPONSE:** Thanks for the observation. *Arabidopsis thaliana* was first mentioned in the abstract and 'Arabidopsis' word was replaced by *A. thaliana* throughout the manuscript.

**Line 83. This should read "*Arabidopsis thaliana*L. (Heynh) wild-type Columbia seed…" It is customary in the Material and Methods to give the full Latin binomial along with the taxonomic authority. And unless you sequenced your Columbia line, you should drop the "0".**

**RESPONSE:** Thanks for the observation. The sentence was modified and now it reads as follows: '*A. thaliana* (L.) Heynh wild-type Columbia seeds'.

**Line 84, and elsewhere. Do not use circle-R, or TM, or other commercial symbols. Those marks exist to protect consumers (not manufacturers) from fraud. Thus if you sell a product that you say contains X circle-R, you are defrauding the consumer if you substitute something cheaper for X. There is no such issue here. In fact you are not selling anyone anything (you are paying).**

**RESPONSE:** Thank you. Changes were made accordingly.

**Line 98. I don't understand the meaning of the word 'power' here.**

**RESPONSE:** Thank you. The word power is not necessary. It was deleted.

**You embedded the figure legends into the results text but not the figures. I think this is awkward. Please put the figure legends with the figures, preferably at the end of the text. Also for the principal component analyses, please simply call your x and y axes "PC1" and "PC2" and put the percentages of explained variance in the figure legend.**

**RESPONSE:** As suggested, figures and legends are together in a separate file uploaded right after the text.

All principal component analyses have been modified and the explained variance is now shown in the legend.

**In figure 3A, the names of some compounds are capitalized but others are not. And "control" is in all caps. This is bizarre. I suggest that none of them should be capitalized, but whatever you do please do the same for all. And note that if you choose to capitalize the first letter, this should be done even for those compounds that start with a number (e.g., "5-Aminovaleric acid"). Also, please put the name of the horizontal axis below, next to the numbers (or put the numbers above, next to the name; the point is, the numbers and the name belong together.**

**Also please remember that the abbreviation for liter is L (not l). And to always put a space between the number and the unit (except when the unit is the degree sign or the percent sign).**

**RESPONSE:** Thanks for the observation. The first letters of all compounds have been capitalized, and the horizontal axis is now next to the numbers. The abbreviation L and the space between the number and the unit was also modified as suggested.

**In Figure 4, there are problems with the y-axes. Many of them have huge numbers. But no units. I think peak area is arbitrary so you could use 1, 2, 3. If it is needed to compare the quantity between states, then you could have the smallest numbers be 1, 2, 3 and the other states relative to that. Also many of the graphs do not start at zero. But some of them do. This is misleading. They should all start at zero. The names of the axes could be in a larger font.**

**RESPONSE:** Thank you for the observation. The y-axis values show the magnitude of the cumulative secretion level of the compound per each stage. Those values are different based on the compounds. Thus, standardizing the values for all compounds will make some compounds hard to asses due to low levels. This set of plots aimed to provide to the reader with a visual aid to see significant differences by compound between the phosphorous treatments.

We think keeping the peak area unit as it is will help the reader to determine the degree of change (of the compound) in response to P amendment as well as to locate the compound that was the most affected by phosphorus amendment.

With respect to graphs not starting at zero: the compounds with statistical significance are properly labeled which will help the reader to see which of those secretion levels are highly responsive to phosphorus addition. We do not think this is misleading and we would rather keep it as it is.

**Reviewer #1: Two minor and three major critics**
**minor:**
**1. Root exudates depending on plant develpment were reported by Keerthisinghe et al. 1998, Plant Cell Environ., 21, 467- 478 and by Neumann et al., 1999, Planta, 208, 373- 382.**

**RESPONSE:** We appreciate your valuable comments. Both papers are very relevant for our work, and we have included references about that work in our manuscript. We modified the abstract to state that: '**However, studies addressing the variability of roots exudates involved in phosphorus solubilization across plant developmental stages remain scarce'. Instead of saying that these studies 'have not been conducted'. Having said this, in contrast with Keerthisinghe et al. (1998) and Neumann et al., (1999), our study showed effects of P supply on non-proteoid plant-roots such as Arabidopsis.

Neumann et al., (1999) support our findings showing that phosphorus mobilizing compounds such as citric acid was predominantly restricted to mature root clusters in the later stages of P deficiency. However, they conclude that this mechanism is 'predominantly confined to proteoid-roots' which is not the case for Arabidopsis as shown in our study. We have incorporated this to the discussion section.

**2. L 278 ff. Misleading since P and carboxylate concetrations must be clearly separated.**

**RESPONSE:** Thank you for the observation. We have rewritten this sentence to better describe how cumulative secretion of different carboxylates vary depending on the treatment (phosphorus level) and to make the distinction between phosphorus and carboxylates. Now the sentence reads as follows: 'Cumulative secretion of malic acid was reduced significantly from 0.3125 to 0.625 mM phosphorus treatments, and then incremented its cumulative secretion for the highest phosphorus treatment (1.25 mM). However, secretion levels of malic acid for the two highest phosphorus treatments (0.625 and 1.25 mM) were below the value for the lowest phosphorus rate (0.3125 mM) (Fig 4A).'

**major:**

**1. Misleading: P starvation response 50 µM P. Cultivation in non buffered systems, agar or solution means that a solution threshald is only valid for a special volume. In soil P is buffered and P starvation values are below 2 µM P , see Föhse te al., 1988, Plant Soil, 110, 101- 109.**

**RESPONSE:** Thank you for the clarification. As per your suggestion, we clarified that plant starvation response to phosphorus is activated at values below 2 µM P. We have added Föhse et al., (1988) reference to the manuscript and the sentence now reads as follows: 'The **three phosphorus levels** used in this study did not induce the plant starvation response, which is generally activated at values below **2 µM** of phosphorus in the soil solution [23].

**2. Decisive for the solubilizing ability of root exudates is the relation between soil solid P and the quatity of exudates (see in detail Gerke, 2015, cited in the ms). Solution concentrations are not an appropriate measure of exudate efficiency. Mesurement of pH changes as a central factor of P mobilization from Ca-P forms?**

**RESPONSE:** We appreciate your valuable comment. We are aware that a combination of factors including carboxylate efflux, the accumulation of carboxylate in the rhizosphere and the chemistry of phosphorus mobilization in the soil phase affect the solubilization ability of root exudates (Gerke, 2015). We acknowledge that we did not take into account these variables, because our study was entirely done in liquid culture to test solubilization ability of the selected compounds, not necessarily efficiency which would require the measurement of abovementioned factors. However, we have added a paragraph in the discussion section that address pros and cons of root exudate studies in liquid conditions (our study).

The paragraph added reads as follows: 'Lastly, root exudates from liquid culture systems allow the determination of exudation rates unaltered by the soil matrix or microbial decomposition if performed under sterile conditions as we did in this study (Oburger and Jones, 2015). However, the quality and quantity of the root exudate profile may be impacted by the nutrient solution culture method (also known as hydroponic methods) (Oburger et al., 2013). Soil-hydroponic hybrids methods for root exudation collection are not exempt of potential physical/physiological perturbances. Thus, sterile nutrient solution culture methods remain especially important to assess temporal dynamics of root exudates.'

**3. The relevance of the results with respect to P solubilization is questionable since the P form is rather unrepresentative and the exudate quantity is rather high.**

**RESPONSE:** Thank you for your observation. It would be helpful if the reviewer elaborates on his comment. Why are the P form is unrepresentative? Is it because calcium phosphate is not necessarily predominant in all agricultural soils? Calcareous soils constitute over one-third of the world's land area. Why you consider the root exudate quantity as high, relative to what? Root exudates concentration varies a lot across P levels, developmental stage and on a per compounds basis in our study.

**Reviewer #2: General comments:**

**The manuscript "Phosphorus assimilation effects by root exuded compounds across plant developmental stages" characterises root exudates across growth stages and P-levels for Arabidopsis. Interesting contrasts were found. The authors select some important exuded compounds and go on to test their ability at solubilising P in soil-analogues individually and together. This was a well written manuscript, with well thought out experiments, executed well leading to interesting results. I really enjoyed reading this manuscript, I learnt a lot that is relevant to my work, and appreciated the work that went into it. I have some general comments that I think would improve the presentation and some of the discussion.**

**1) I think in the materials and methods the presentation of the PCA is sometimes over complicated, and could be made simpler. My detailed comments below would address this.**

**RESPONSE:** Thanks for the valuable observations. Detail corrections regarding the PCA plots have been addressed in each of your comments below.

**2) The presentation of the PCA results could be improved. I think showing all the data with no groupings used on the PC-axis (with different colours and marker styles and legends!) would make it much clearer an. This could come at the expense of some of the plots where treatments are grouped. Also make it clear if a PCA is done per grouping or not. Again, many of my detailed comments try and address this.**

**RESPONSE:** Thanks for the valuable observations. Detail corrections regarding the PCA plots have been made and addressed in each of your comments below.

**3) The discussion would be improved by quickly pointing out the agricultural/ecological significance (or lack of) of the results. For example 7mM of acids order of magnitude more than what is found in the rhizosphere. See the Eva Oburger papers I referenced. Hence the solubilisation seen here might not appear in the field. One of our group's papers found that P solubilisation by a single root exuding citrate at a realistic rate actually made no difference to P uptake by the root https://doi.org/10.1007/s11104-019-04376-4 (dont feel you have to reference this). It also might be worth pointing out how similar NBRIP media is to soil, solubilisation in this media might not be the same as soil. Also see my detailed comment regarding your proposed mechanism number 1.**

**RESPONSE:** Thank you for the observations. We have added a paragraph that discusses the relevance and ecological significance of organic acids as phosphorus mobilizers and the relevance of the root exudate analysis in liquid cultures.

The new paragraph reads as follows: ‘It has been estimated that organic acids constitute 5 to 10 % of the total organic carbon in the soil solution. The concentration of organic anions measured in the soil solution usually range from 100 nM to more than 580 uM in the rhizosphere of cluster roots (Jones, 1998). However, millimolar concentrations of organic anions are likely required in the soil solution to effectively increase soluble P concentration especially in calcareous soils (Strom et al. 2005; Ryan and Jones, 2001). Strom et al. (2005) tested three organic acids (citrate, malic and oxalate) and a wide range of concentrations (1 mM to 100 mM) to evaluate its effects on the mobilization of phosphorus in calcareous soil. The results showed that the phosphorus mobilization of the tested compounds had a low efficiency and its effect varied depending on the type of organic acid, compound concentration, and pH. Further, due to the low phosphorus mobilization efficiency of those compounds it is still argued if the benefit of releasing large amounts of organic acids into the soil will exceed the cost of carbon lost by the plant, which can be seen as an unnecessary trade-off (Strom et al. 2005). However, low efficiency organic acids can be particularly important in phosphorus mobilization for calcareous soils with a limited phosphorus availability for plants. Finally, our evidence supports the above-mentioned hypothesis, that plants release a combination of compounds with different phosphorus-solubilizing efficiencies, at specific stages of growth, to deal with particular phosphorous needs.’

**Sorry, I have a lot of detailed comments, however many of them are pointing out the same thing and many are complimentary. I hope they aren’t too hard to deal with.**

**Detailed comments:**
**L25: roots->root**

**RESPONSE:** Thank you. Please see correction in line 28.

**Abstract is clear and well written.**

**The paragraph starting line 67 doesn’t segway smoothly into that starting on 73 because root exudates have been shown to be less important for good P conditions (not to say that the paragraph on L67 isnt useful introductory information). Calling back to the ‘legacy P’ argument here would make it smoother.**

**RESPONSE:** Thank you for the observation. We considered your suggestion and have improved the transitioning sentence between the two paragraphs. Now it reads as follows: ‘Due to the variation of phosphorus demand during the plant’s lifetime, it becomes necessary to fully understand fluctuations of root exudates as a means to solubilize the phosphorous present in the P legacy of the soil.’

**L90: ‘gradient’ isn’t the best word here. It makes it seem like the growth media each plant is grown in has a gradient of P conditions in it. Is ‘The low P conditions used in this study….’ Better?**

**RESPONSE:** The authors used gradient to refer to the three concentrations of phosphorous used in the study. For clarity, we changed it to: ‘The three phosphorus levels used in this study did not stimulate the plant starvation response, which is generally activated around 2 μM of P [23].’

**L129: '…which compounds had the highest correlation with these components through the magnitude of the variance'. 'correlation' is unclear here. Do you mean which two original basis vectors (compounds) explained the most variance? This would be an easier way of explaining it.**

**RESPONSE:** Thank you for the observation. No, in this sentence (now line 140) we refer to the magnitude of the loadings of the compound (variance of the compound secretion level) not to the PCA explained variance. We elaborate on the meaning in more detail in the two following sentences in the manuscript.

**L133: 'The largest…' Is it not that these compounds explain the most variance? Its best to write these in terms of what percent of the original variance they explain (before dimension reduction).**

**RESPONSE:** Here we refer to the variance of the individual compounds based on the secretion level (loadings). Compounds with the largest loadings drive the PCA variance and therefore were selected for further testing. Please see line 131 to 146 for more detail.

**L134: differential isn't the right word here.**
**I think the end of this paragraph can be explained in a simpler way by saying: "We determined which compounds explained the most variance for each P level and growth stage. The contrasts in these 'most principle compounds' over growth stage and P level indicated differences in exudation depending on these factors" Or something like this.**

**RESPONSE:** Thank for your suggestion. We have modified the sentence for clarity and now it reads as follows: 'this method allowed us **to determine** which compounds **explained most of the variance** across the fertilizer levels and for each of the plant's growth stages.'

**L144: This needs more explanation this is quite a large range, how did you 'adjust' the concentrations? The reader needs to know how this is done to interpret the results. Did you take concentrations from these references to calibrate your values? Due to the variability in reported exudate concentration due to sampling technique (https://doi.org/10.1016/j.rhisph.2018.06.004) 'exudate concentration' is relative to the study. Probably needs a mention in the discussion.**

**Side note: Could be worth mentioning that you didn't adjust the concentrations for the PCA, when I first read it I thought you had.**

**RESPONSE:** Thanks for your observations. As stated in the text, we used a range of concentrations reported in the literature as a reference to select the concentration of the compounds tested in this study. The word 'adjust' has been replaced by the word 'select' for clarity.

As you well pointed out, the range of concentrations of this compound is wide. We cited 3 papers that support that the concentrations used in our experiments are present in the rhizosphere (Jones, 1998; Veneklaas et al., 2003 and Ström et al., 2005). In addition, we have added a paragraph in the discussion where we elaborate on the concentrations of compounds selected for this study (please see previous answer to your comment) and included the paper that you suggested: Oburger and Jones, 2018.

**L152: What concentrations did you add to test the P solubilising ability of the compounds? Were they comparable to rhizospshere concentrations? Knowing this puts this into an ecological context**

**for the reader. Is the visual inspection for solubility standard? Add a reference if so. Otherwise explain the reasoning.**

**RESPONSE:** Thank you for the comments. We direct the reviewer to the methodology sub-section (149-160) where the rationale for selecting the concentrations to test P solubilization and the actual concentrations are stated; as well as the paper showing the efficacy of the qualitative technique used in our study. Please see reference number 30 in the reference list (Nautiyal et al., 1999. *FEMS microbiology Letters*, *170*(1), 265-270).

**Table 1: were they diluted to 100mM before PCA? Would this effect PCA? (see my side note above). 100mM is a really high concentration for most root exudates in the rhizosphere no? I know for most organic acids and amino acids I would expect micro molar concentrations in the rhizosphere, see https://doi.org/10.1016/j.envexpbot.2012.11.007**

**RESPONSE:** Thanks for the observation. Indeed, PCA show total root exudates as a response to phosphorus levels. We agree, 100 mM is in the higher end of the concentrations. This concentration was used only in the qualitative experiment (Petri dish). Several lower concentrations were used as well, and we decided to use 100 mM for this qualitative analysis which aimed to provide an initial visual tool to narrow down a long list of compounds that could solubilize P. Please note that we reduced this concentration to 7 mM and the compounds still showed phosphorus solubilization.

**L171: again this a high concentration for the rhizosphere, I would expect a discussion on the ecological relevance later.**

**RESPONSE:** This question was addressed above.

**L181: I think ‘cumulative’ effect is better than ‘additive’. Additive makes it seem like you could add up the effects individually to determine to summed effect.**

**RESPONSE:** Thank you. We replaced the word additive for the word ‘cumulative’ to better describe the meaning.

**L200: unclear what ‘distribution’ means here. After looking at FigS1 we see it’s the groupings after plotting on the first two principle components. This needs to be made clearer. I think the author means the separation of growth stage and P treatment by PCA persists after throwing away the non-annotated compounds. Which implies the non-annotated compounds explained little of the variance, which I believe justifies the author’s conclusion. Were the principle components determined twice i.e. before and after throwing away the non-annotated compounds? FigS1 could use a legend saying what the other colours are.**

**RESPONSE:** Thank you. For clarity, the word distribution has been replaced by the word “grouping”. In addition to indicating that the PCA was conducted with the data after removal of the non-annotated compounds, we have modified this sentence and now it reads as follow: ‘The variability in our data **after subtracting the non-annotated compounds** was analyzed using a principal component analysis (PCA) where variability of component 1 (PC1) accounted for 29.8%, while component 2 (PC2) accounted for 21.7%.’

PCA from figure 1 is identical than supplementary figure 1. Both depict the overall dissimilarity of compound groups across phosphorus levels and growth stages. Both figures show detailed information in the legend now.

**Fig 1A also needs a legend.**

**RESPONSE:** Thank you. Figure 1A has now a legend with the suggested information.

**In Fig 1A did you group by developmental stage, do PCA then plot? If this is the case, the % variance explained by the PCs reported in line 203 isnt correct.**

**RESPONSE:** Thank you for the comment. For figure 1 as well as for supplementary figure 1, no grouping was performed before constructing the PCA. The complete data set from three developmental stages and 3 levels were plotted and analyzed at the same time without previous modification. As suggested, the explained variance of figure 1A was added to the legend. For more detail, please see line 132-144.

**Same goes for Fig 1B but by P level? The different percent of variance explained by components suggests this is the case. Need legend for Fig 1B also.**

**RESPONSE:** In figure 1A, we present the three plant stages and three phosphorus levels (all data). Figure 1B presents data for three phosphorus levels within the vegetative stage only, and figure 1C presents the three phosphorus levels within the vegetative stage only. The explained variance for each PCA has been added to the figure legend.

**I would use colours to indicate developmental stage and stars/dots/crosses to indicate P-levels (or vice versa). This and a legend would make fig 1 a lot easier to understand.**
**Fig 1A caption is wrong "201 annotated compounds with proper identification detected using GC-MS are plotted on the Principal Component Analysis (PCA) graph". The compounds aren't plotted. The compounds are the 201-axis, the dimension is then reduced to 2-axis with PCA and the locations of the growth stages are plotted.**

**RESPONSE:** Thank you for the clarification. Your observation is technically correct. We have replaced the word plotted. Now, the sentence reads as follows: '201 annotated compounds with proper identification detected using GC-MS **were analyzed using a** Principal Component Analysis (PCA) graph.'

With respect to editing the colors. The phosphorus levels and growth stages are color-coded, and the information related to each treatment is explained in the legend. We would rather keep the graphs as they are currently.

**L205: "We determined that the plant's developmental stage was responsible for the separation of the compounds in three marked groups" to convince the reader that its not P level you would need to not group by anything and plot all the data with stars/dots/crosses and colors.**

**All these figures desperately need legends (Fig1, S1, S2) and clearly state if there is a PCA per grouping.**

**RESPONSE:** Thank you. This comment was addressed in the previous answers.

**L230: explained the most variance.**

**RESPONSE:** Thank you. Following your suggestion, we have corrected this sentence and now it reads as follows: 'For each of these treatments, we found the five top compounds that explained the largest proportion of the variance in the principal components (Table 1).'

**L231: 'correlation' isn't really defined here. Its more that the compounds which point in the most similar direction to the two principle components, I would get rid of the bit after the comment.**

**Change this sentence to: For each of these treatments, we found the five top compounds that explained the largest proportion of the variance.**

**RESPONSE:** Thanks. We added this suggestion in the previous response.

**Figs S3-S4 are really intresting! Great results**
**Same goes for Fig 2 and 3A**

**RESPONSE:** Thank you.

**L254: does the 1.75 7 and 28 mM mean that the sum of the concentrations or each have that concentration? Fig 3 caption makes this clear but also make it clear in the manuscript.**

**RESPONSE:** Good observation. Each compound added to the pool had 1.75 mM, 7 mM and 28 mM concentration. We have added this detail in the manuscript. As explained in the methodology section line 189-195: 'In order to determine the potential cumulative effect of 3-hydroxypropionic acid, malic acid, nicotinic acid, and glutamic acid they were combined and the available phosphorus (mg $l^{-1}$) was determined by OES-ICP. A phosphorus gradient that included the previous tested concentration (7 mM per compound), a higher (28 mM per compound) and a lower (1.75 mM per compound) concentration was tested in order to compare if the combination of compounds would equal or surpass the effect of a single compound. Each compound added to the pool had 1.75 mM, 7 mM and 28 mM concentration. Thus, the combination effect of four compounds were tested in a liquid NBRIP medium.'

**L255: The 1.75M is really interesting, 7mM of them mixed does worse than 7mM of any individually**

**Again, really interesting results with Fig 4, very hard to interpret though there is lots going on.**

**RESPONSE:** Each compound has a different molarity; therefore we cannot say that the 4 compounds at 1.75 mM add up to 7mM. We conclude that 1) compounds in combination have an additive effect, and that 2) the addition of one does not inhibit the effect of the other.

**L300: …'where optimum conditions are scare' -> where nutrients are scarce ??**

**RESPONSE:** Thank you. We have corrected this sentence and now it read as follows: 'Thus, we hypothesized that at this growth stage roots did not respond to phosphorus fertilization. *A. thaliana* is considered a plant that can thrive in marginal soils where optimum **nutrient** conditions are limited.'

**L304: These papers might be useful for this discussion on maize need for P early on with respect to yield: https://doi.org/10.1007/s11104-011-0814-y. https://www.nrcresearchpress.com/doi/pdfplus/10.4141/P00-093) Take them or leave them**

**RESPONSE:** Thank you for the recommendation. As suggested, those two relevant papers were included one in the introduction section (Grant et al., 1999) and the second in the discussion section (Nadeem et al., 2011).

**L309: 'similarity' do you mean they grouped close together in the PSA? To talk about similarity you would need to use a norm between the treatments.**
**RESPONSE:** Thanks for the observation. We are referring to visual similarity. We have corrected by adding: **'**In the vegetative stage, the root exudates at 50% and 100% phosphorus showed greater visual similarity in the PCA than the exudates at 25% phosphorus, evidencing an initial sensing from the plant in response to its phosphorus demand.'

**L323: I think your mention of other functions of exudates is a good explanation and something other authors overlook.**
**RESPONSE:** Thank you.

**346: that 'the' shouldn't be there**

**RESPONSE:** Thank you; 'the' was removed.

**L351: new paragraph**

**RESPONSE:** Thank you.

**L352: mechanism 1. Your results suggest there is no synergy (in fact the opposite) in terms of P solubilisation by root exudates, see my comment about L255, at least for the 4 acids you picked out. But this doesn't disprove your mechanism: I think I read somewhere that certain exudates solubilise P from certain soil surfaces, Al oxides, Fe oxides etc (possibly a paper by Gerke about citrate and malate?) Maybe this is why they exude a mixture of acids? Did the soil-analogue you used have this variability in mineral surfaces which soil has?**
**Nonetheless, I think this deserves some more discussion.**

**RESPONSE:** We addressed this question in comment L255 above.

**L357. I like point 2, I had never heard of this before.**
**RESPONSE:** Thank you.

**I don't think Table S1 is mentioned in the manuscript**

**RESPONSE:** Table S1 show data that is already presented in supplementary figure 3 and 4 in more detail. Therefore, is was deleted it.
