# Supplementary material for: Role of root exudates on assimilation of phosphorus in young and old Arabidopsis thaliana plants
Source: PLoS One. 2020 Jun 3;15(6):e0234216. doi: 10.1371/journal.pone.0234216 (PMC7269232; doi:10.1371/journal.pone.0234216)
Supplement: Attachment — Submitted filename: Reviewersresponses-0518.docx [file pone.0234216.s007.docx]

**Reviewers responses**

Editor comments:

The second comment concerns figure 3B where you check the solubilizing activity of combinations of the key compounds. The problem here is that you test the single compounds at 7 mM (Fig. 3A). But you test the combinations at that concentration, and also at lower and higher concentrations. I do not see how the higher and lower combinations can be interpreted without also showing values for the single compounds at each concentration. Without that data, I suggest adding the 7 mM combined results as the last bar of figure 3 A and deleting the data for the other combinations (unless you happen to have data for single compounds at the alternate concentrations). By the way, given that the combination solubilized about the same amount of phosphate as did 3-hydroxyproprionate alone, I don’t see how you can even think about an additive effect.

**RESPONSE: We agree with the editor. Following your recommendation, the 7 mM combined results from figure 3B was added to figure 3A. Figure 3B was removed. Legend of figure 3 was modified accordingly. Results and methods section were also updated accordingly.**

Reviewer #2:
Thank you for considering my comments so thoughtfully in the response. I believe the authors have answered the vast majority of my comments, I only have one outstanding query regarding the following in the response document:
'''
L255: The 1.75M is really interesting, 7mM of them mixed does worse than 7mM of any
individually
Again, really interesting results with Fig 4, very hard to interpret though there is lots going on.

RESPONSE: Each compound has a different molarity; therefore we cannot say that the 4 compounds at
1.75 mM add up to 7mM. We conclude that 1) compounds in combination have an additive effect, and
that 2) the addition of one does not inhibit the effect of the other.
'''
I think concluding there is an ‘additive effect’ is a bit miss leading. Here is the standard definition of an additive affect [https://www.dictionary.com/browse/additive-effect](https://nam01.safelinks.protection.outlook.com/?url=https%3A%2F%2Fwww.dictionary.com%2Fbrowse%2Fadditive-effect&data=02%7C01%7Ch.Pantigoso_Guevara%40colostate.edu%7Cd67c8642f2b8479ea00108d7f358ecc0%7Cafb58802ff7a4bb1ab21367ff2ecfc8b%7C0%7C0%7C637245435588206803&sdata=UxQuUAcGx4S9wYp22YeY7uAEQwohsEr3pDq8AwC15jQ%3D&reserved=0). The sum of 7mM individually is much more than 7mM combined (I use the 7mmol case now so there is no issues with the molarity. Both cases has 7mM of each.) thus it is not an additive system. This is of course because in the individual cases are done in separate assays and they solubilise the lightly-bound P pool 4 times, while the combined is done in one assay. I think if you want to convince the reader that they are ‘additive’ (and later ‘synergize’ L348 in the discussion) you need to define exactly what it means for the current system to be additive or synergize. Possibly the current experiments and media aren’t suitable to make this conclusion. As I mentioned before, I suspect the compounds ‘synergise’ (I define this to mean to do better than the sum of its parts) when the media has a range of minerals that buffer P and are attacked more efficiently by certain exudates. For example (this is entirely hypothetical) assume the soil is made up of 50% Al-oxide and 50% Fe-oxide and nicotinic acid solubilises P from Al-oxide very effectively but badly from Fe-oxide and malic acid vice-versa. In this case the two compounds would certainly synergise. As I mentioned before I think there is papers about this but I cannot remember, the other reviewer Prof Gerke is likely to know about this and can point you in the right direction. I note the paper “Does the combination of citrate and phytase exudation in Nicotiana tabacum promote the acquisition of endogenous soil organic phosphorus?” which you cite for the synergy of exudates for P solubilisation is an experiment done in soil not NRIB.

I do not think more experiments or analysis is needed as this is a minor result in the overall paper, but a more careful discussion of this is needed in liight of the results.
I do agree with conclusion that the addition of one does not inhibit the other which is a good result in itself.

**RESPONSE: We appreciate your follow-up comments. Due to a similar concern by the editor we have modified Figure 3 and made changes to the methods and results sections to address in the best way possible your comment. Please see below:**

1. **We incorporated the 7 mM combination from figure 3B as the last column of figure 3A. We removed figure 3B. We agreed that not having single data for every compound with different concentrations, including 1.75 and 28 mM, make difficult to assess the effects. The result (line: 245-254) and method sections (line: 186-193) were modified accordingly to reflect these changes.**
2. **With respect to the citation: we were not able to find a similar result under the same conditions used in our study (NBRIP media). We kept the citation because we believe this paper is relevant for our discussion. However, to address your concern, we have clearly stated that the conditions from that finding are different that ours. Now it reads as follow: ‘**However, this study was performed under soil conditions and not using liquid NBRIP media”. In addition, it is clearly stated in this paragraph that these are hypotheses that will warrant experimental testing.

Detailed comments:
L187 (on tracked document): Should ‘A phosphorus gradient’ here instead be a ‘compound gradient’?

**RESPONSE: Good observation. The word gradient was replaced by ‘compound mixture’. Now the sentence read as follow:**

*‘A compound mixture that included the previous tested concentration (7 mM per compound) was tested in order to compare if the combination of compounds would equal or surpass the effect of a single compound’*

I think when describing the method for the cumulative experiment, you should make it clear whether the mixtures were all added to 5 mL of liquid NBRIP so that the same amount of P is the same as the individual experiment. Currently it is unclear.

**RESPONSE: Thank you. We added this information to the result section. Now it reads as follow:**

‘…. Briefly, 35 μL (100 mM) of each compound was added to 5 mL liquid NBRIP medium resulting in a final concentration of 7mM. Each compound added to the pool had 7 mM.’
